# Sorafenib, a clinical kinase inhibitor, attenuates *Streptococcus pneumoniae* pathogenesis and reduces disease progression *in vivo*

**DOI:** 10.1128/mbio.00618-26

**Published:** 2026-06-16

**Authors:** Joel Abraham, Aswathy C. Sagilkumar, Himani Dhyani, Charmi M. Panchal, Shaheena Aziz, M. K. Priyadatha, Aan Ruth, Keerthana Bhaskaran, Sivakumar Krishnankutty Chandrika, S. Shaima, Rosemol Varghese, Ayyanraj Neeravi, Balaji Veeraragavan, Nagarjun Narayanaswamy, Sandhya Ganesan, Karthik Subramanian

**Affiliations:** 1 Host-Pathogen Laboratory, Pathogen Biology Division, BRIC-Rajiv Gandhi Centre for Biotechnology (BRIC-RGCB) 81754, Thiruvananthapuram, India; 2 Regional Centre for Biotechnology, Faridabad, India; 3 School of Biology, Indian Institute of Science Education and Research 193159, Thiruvananthapuram, India; 4 Bioinformatics Facility, BRIC-RGCB 81754, Thiruvananthapuram, India; 5 Transdisciplinary Biology Program, BRIC-Rajiv Gandhi Centre for Biotechnology 81754, Thiruvananthapuram, India; 6 Department of Clinical Microbiology, Christian Medical College 196380, Vellore, Tamil Nadu, India; The University of Mississippi Medical Center, Jackson, Mississippi, USA

**Keywords:** *Streptococcus pneumoniae*, serine threonine kinase, StkP, sorafenib, antimicrobial resistance, drug repurposing

## Abstract

*Streptococcus pneumoniae* is an opportunistic respiratory commensal bacterium and leading cause of pneumonia, meningitis, and sepsis, resulting in over a million deaths annually, particularly in children aged under 5. The rapid spread of macrolide-resistant strains led the WHO to designate *S. pneumoniae* as a priority pathogen that urgently requires alternative therapeutic strategies. Here, we identified the FDA-approved cancer drug sorafenib to show a dose-dependent, broad-spectrum efficacy against many pneumococcal serotypes, including multidrug-resistant clinical strains. *In silico* screening and molecular dynamics simulations indicated potential interaction with the catalytic cleft of the pneumococcal serine/threonine kinase StkP, a central regulator of cell division and peptidoglycan synthesis. *In vitro* kinase assays using purified recombinant StkP kinase domain and p-Thr-specific immunoblotting revealed dose-dependent inhibition of kinase activity by sorafenib in pneumococci. Ectopic expression of StkP partially rescued growth inhibition by sorafenib, and the direct interaction was assessed by isothermal titration calorimetry, suggesting StkP as one of the potential targets in *S. pneumoniae*. Sorafenib-treated bacteria showed abnormal morphology, increased membrane permeability, enhanced complement C3 deposition, and reduced adherence and invasion into lung epithelial cells without significant host cytotoxicity. Serial passaging of bacteria *in vitro* with sorafenib suggested low resistance potential. *In vivo*, sorafenib administration at 10% of the clinically relevant dose delayed mortality and significantly reduced bacterial burden in a murine pneumonia model, supporting its further preclinical development for therapeutic intervention.

**IMPORTANCE:**

*Streptococcus pneumoniae*, a WHO priority pathogen and causative of pneumonia, meningitis, and sepsis worldwide, has developed resistance to several commonly used antibiotics, limiting available treatment options. In this study, we show that sorafenib, a drug currently approved for cancer treatment, can also inhibit the growth and disease-causing ability of *S. pneumoniae*, including clinical strains. Our findings reveal that sorafenib interferes with a key bacterial regulatory enzyme that controls cell division and cell wall formation, exposing a previously underexplored vulnerability in this pathogen. Because sorafenib is already clinically approved, this work highlights the potential of drug repurposing as a faster route to identify new antimicrobial therapies. More broadly, our results demonstrate that bacterial kinases represent promising targets for developing next-generation treatments against key bacterial pathogens.

## INTRODUCTION

*Streptococcus pneumoniae* (pneumococcus) is a gram-positive human pathogen responsible for many invasive (pneumonia, meningitis, and sepsis) and non-invasive (otitis media) diseases, causing an estimated 1.6 million deaths annually (1, 2). The global burden of pneumococcal infections is substantial, particularly affecting young children, the elderly, and immunocompromised individuals. Notably, *S. pneumoniae* is the highest causative of mortality among young children post-neonatal to age 4 years (3). The clinical burden is exacerbated by the rapidly rising antimicrobial resistance that poses significant challenges to effective treatment and disease management (4). Over the past few decades, the emergence and spread of multidrug-resistant pneumococcal strains has been attributed to the widespread and often indiscriminate use of antibiotics, as well as the bacterium’s remarkable ability to acquire and disseminate resistance genes through horizontal gene transfer (5). Resistance to currently used antibiotic classes such as beta-lactams, macrolides, and tetracycline has been reported, and multidrug-resistant strains are emerging worldwide (6, 7). In 2024, the WHO classified the macrolide-resistant *S. pneumoniae* as one of twelve priority pathogens in urgent need of new therapeutics.

Targeted inhibition of conserved bacterial proteins is a viable broad-spectrum antibacterial strategy and may impose less selective pressure for resistance (8, 9). Several bacterial factors contribute to the pathogenicity of *S. pneumoniae*, including the polysaccharide capsule, surface proteins, and proteins involved in host interactions (1). Among these, the serine/threonine kinase protein, StkP, is a potential therapeutic target. StkP is a key regulator of pneumococcal physiology and virulence, playing crucial roles in cell division, cell wall biosynthesis, competence, and stress response (10). In *S. pneumoniae*, StkP has been shown to phosphorylate multiple substrates, including cell division proteins (FtsZ and DivIVA), cell wall biosynthesis enzymes (MurC and GlmM), and transcriptional regulators (RitR) (10, 11). Through these interactions, StkP modulates various cellular processes that are critical for pneumococcal survival and virulence. Genetic disruption of StkP attenuates virulence in animal models, impairs cell division, diminishes biofilm formation, and increases sensitivity to environmental stressors, all of which contribute to decreased virulence (10, 12). Several characteristics make StkP an attractive target for therapeutic intervention. First, its conservation across pneumococcal strains and presence of homologous protein, PknB, in *Staphylococcus aureus* (13) and *Mycobacterium tuberculosis* (14) suggests that targeting StkP could provide broad-spectrum activity against many priority pathogens. Second, the multifaceted role of StkP in bacterial physiology implies that its inhibition could potentially show therapeutic efficacy.

Here, we screened 38 host-targeted kinase inhibitors against the pneumococcal StkP kinase domain and identified sorafenib as the lead compound, which is currently an FDA-approved drug for hepatocellular carcinoma (15). Although sorafenib is clinically used to inhibit VEGFR, PDGFR, and RAF kinases in the treatment of hepatocellular carcinoma, a recent study reported its activity against methicillin-resistant *S. aureus* (MRSA) by inhibiting menaquinone biosynthesis and protein secretion, without a defined kinase target (16). Before developing sorafenib or its derivatives for antimicrobial therapy, it is crucial to elucidate the mechanisms through which it demonstrates antimicrobial activity against different pathogenic bacteria. In this study, molecular dynamics simulations predicted sorafenib to interact with the StkP active site in competition with the natural substrate DivIVA. We then characterized antimicrobial activity through kinetic growth assays, membrane permeability studies, complement-mediated serum killing, and epithelial cell invasion assays. The efficacy of sorafenib against multidrug-resistant clinical isolates was assessed, and inhibition of the serine/threonine kinase StkP was validated through *in vitro* kinase assays and p-Thr immunoblotting in treated bacteria. The interaction between StkP and sorafenib was studied by isothermal titration calorimetry, and the functional relevance was confirmed via genetic rescue experiments. Serial passaging of bacteria for 31 generations with sorafenib revealed no significant change in the minimal inhibitory concentration, underscoring its low propensity for resistance development *in vitro*. The efficacy of sorafenib was further validated in a mouse infection model.

## RESULTS

### Sorafenib interacts with the catalytic site of pneumococcal StkP *in silico*

The pneumococcal eukaryotic-type kinase StkP phosphorylates downstream substrate proteins like DivIVA (17, 18), FtsZ (19), MapZ (LocZ) (20), MacP (21), and KhpB (Jag/EloR) (22, 23) that are collectively involved in peptidoglycan regulation for the cell wall homeostasis (10). The N-terminal region of StkP protein consists of a catalytically active, cytosolic kinase domain (hereafter referred to as StkP-KD) spanning to 273 amino acids, followed by a juxtamembrane region (274–344), transmembrane domain (345–363), and four PASTA (for penicillin-binding protein and serine/threonine kinase associated) domains (364–434, 435–504, 505–577, and 578–651) (Fig. S1A). StkP-KD consists of a catalytic loop, activation loop, P-loop, and P + 1 loop, which are collectively involved in the phosphorylation activity of StkP (24). Since there is no available crystal structure for StkP or the kinase domain, the three-dimensional structure was predicted using the neural network-based model, AlphaFold (25) (Fig. S1B), and the generated structure was validated using Ramachandran plot analysis (Fig. S1C). Additionally, the StkP-KD structure was superimposed with the available crystal structures of homologous kinase proteins, PknB (*S. aureus*), PknA, and PknB (*M. tuberculosis*) with 47.84%, 42.17%, and 41.26% sequence identities, respectively (Fig. S1D). The residues in the catalytic loop, Val-133 to Asn-141, and ATP-binding P-loop of StkP-KD were highly conserved in all three bacterial species. Pneumococcal StkP-KD showed a high structural similarity with that of Staphylococcal and Mycobacterial PknA and PknB proteins, with root-mean-square deviation (RMSD) values of 1.053, 1.316, and 1.006 Å, respectively (Fig. S1E).

Next, we tested the similarity of pneumococcal StkP-KD with human kinases at the sequence and structural levels. We found that there was only 30% overall sequence similarity (Fig. S2A), but catalytic residues, His-134, Arg-135, Asp-136 (HRD motif), Leu-137, and Asn-141 in the catalytic loop, as well as glycine-rich P-loop in the ATP-binding site, were conserved (Fig. S2B). Structural homology modeling revealed the conservation of β-strand-loop-β-strand motif in the ATP-binding pocket and catalytic loop (Fig. S2C). This prompted us to hypothesize that human kinase inhibitors could be repurposed against pneumococcal StkP.

We screened 38 host-targeted kinase inhibitors (Table S1), previously reported to exhibit activity against liver-stage malaria infection (26), for potential binding to pneumococcal StkP. Molecular docking analysis of the molecules with StkP-KD was performed using the Glide extra-precision scoring function on the Schrödinger suite (27). The top two compounds were Dasatinib and Sorafenib, with docking scores of −8.304 and −8.279 kcal/mol, respectively, which were chosen for experimental validation (Table S2). Growth inhibition analysis showed that sorafenib completely inhibited the growth of *S. pneumoniae* serotype 4, TIGR4 strain, compared to dasatinib at 10 µM (Fig. S2D).

**Fig 1:**
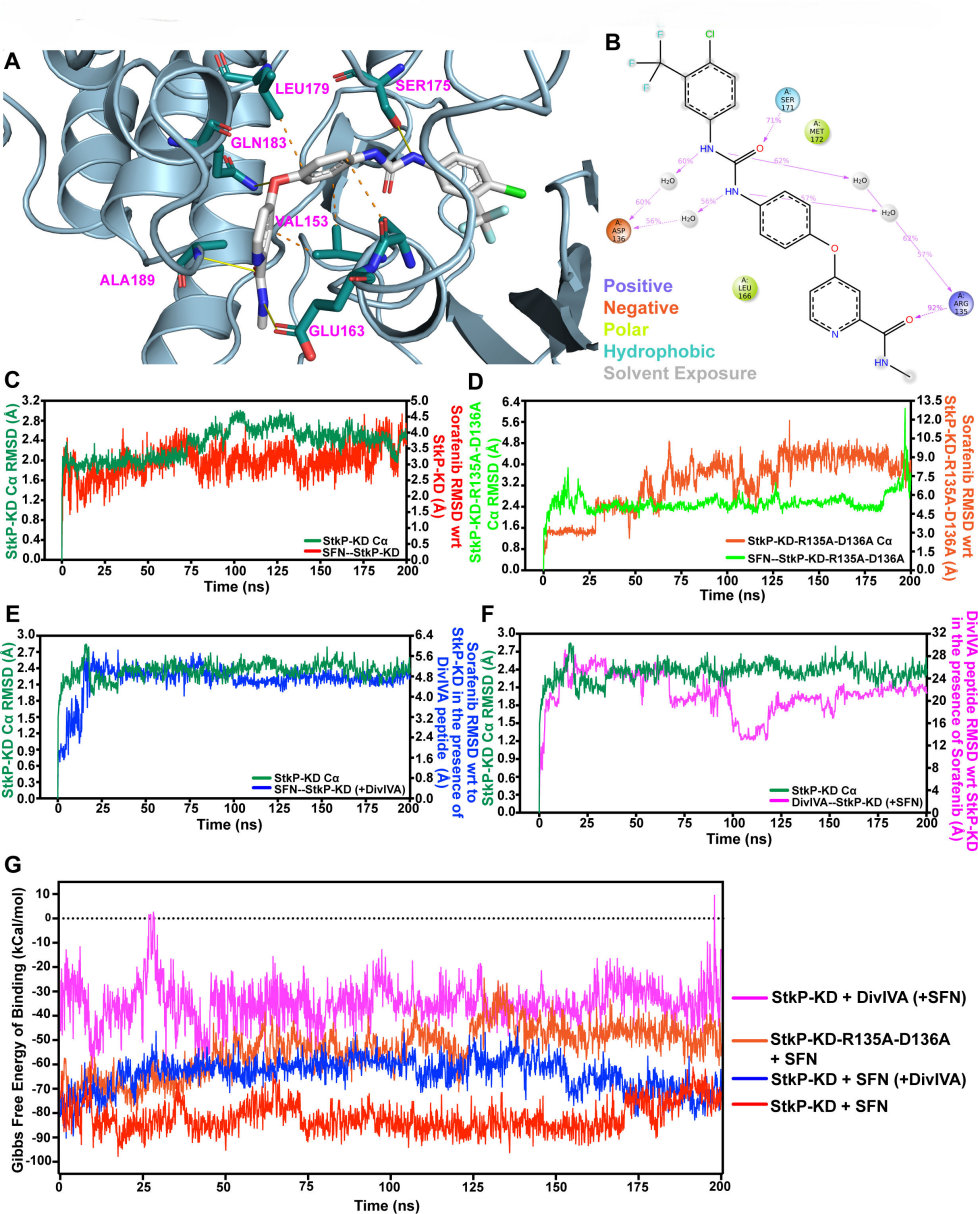
Sorafenib interacts with the catalytic site of pneumococcal StkP *in silico*. (**A**) Docked pose of sorafenib (SFN) at the catalytic site of pneumococcal StkP-Kinase Domain (StkP-KD), showing the major amino acids involved in initial hydrogen bonding (yellow straight lines) and hydrophobic (orange dotted lines) interactions. (**B**) Contact diagram showing the interactions between SFN and StkP-KD during a 200 ns molecular dynamic simulation. Amino acids that interacted for more than 30% of the total time are shown here. (**C and D**) RMSD analysis of the structural fluctuations of (**C**) StkP-KD wild-type and (**D**) StkP-KD R135A/D136A α-carbon backbone and SFN with respect to the proteins. (**E and F**) RMSD analysis of (**E**) StkP-KD α-carbon backbone and SFN with respect to StkP-KD in the presence of DivIVA peptide and (**F**) StkP-KD α-carbon backbone and DivIVA peptide with respect to StkP-KD in the presence of SFN. (**G**) Gibb’s free energy of binding of SFN to wild-type StkP-KD and StkP-KD R135A/D136A showing the importance of residues Arg-135 and Asp-136. The relative binding energies of SFN and DivIVA peptide to StkP-KD in a triple molecule simulation are shown, indicating lower binding energy for StkP-SFN compared to StkP-DivIVA peptide in the presence of SFN.

Docking studies of StkP-KD with sorafenib showed stable binding to the catalytic cleft of the protein (Fig. 1A). A 200 ns molecular dynamics simulation of the docked complex showed consistent hydrogen bonding and hydrophobic bonding interactions with the catalytic residues, Arg-135 and Asp-136 (Fig. 1B; Fig. S3A and B). Furthermore, RMSD analysis of the complex over the 200 ns molecular dynamics trajectory exhibited minimal deviation, corroborating the formation of a stable complex (Fig. 1C and Videos S1 and S2). RMSD measures the average deviation of atomic positions of protein and ligand in a complex from the initial conformation with respect to the backbone C_α_ atom over the period of simulation, with lower values typically within 2–3 Å, indicating structural stability of the complex. To test the involvement of the catalytic loop residues, Arg-135 and Asp-136, in binding to sorafenib, the simulation was performed with both residues mutated to alanine. The mutations abolished interaction with the StkP-KD catalytic loop (Fig. S3C through E), resulting in ~2.5-fold higher RMSD value for sorafenib compared to the wild-type sequence (Fig. 1D), which eventually caused the sorafenib to drift away from the binding pocket (Video S3). DivIVA is a substrate of StkP, which is phosphorylated at the Thr-201 position (28), and plays a vital role in proper septum formation and nucleoid segregation during cell division. Due to the absence of a crystal structure, the structure of DivIVA peptide was modeled using AlphaFold, and a nine amino acid peptide spanning the Thr-201 residue was used in competition with sorafenib to test if it can compete with sorafenib for the binding pocket of StkP-KD. DivIVA peptide was placed near the catalytic area of StkP-KD along with sorafenib. A 200 ns simulation showed that the peptide could not replace sorafenib in the binding pocket and eventually moved away from the active site (Videos S4; S5). While sorafenib showed persistent interactions with StkP catalytic residues in the presence of DivIVA (Fig. S4A and B), DivIVA did not interact with the catalytic site in the presence of sorafenib (Fig. S4C and D). Moreover, both StkP-KD and sorafenib showed a stable RMSD throughout the simulation period, whereas DivIVA displayed high RMSD fluctuations at several points of the simulation period with ~4-fold final RMSD value compared to sorafenib, indicating unstable interactions in the presence of sorafenib (Fig. 1E and F). We also performed Molecular Mechanics with Generalized Born and Surface Area solvation (MM/GBSA) analysis to calculate the free energy of the binding of sorafenib in simulation systems containing sorafenib alone and in the presence of DivIVA or StkP-KD R135A/D136A double mutant. MM/GBSA analysis showed lower free binding energy of sorafenib with StkP compared to DivIVA (Fig. 1G). Besides, mutation of Arg-135 and Asp-136 residues to alanine also increased the free energy of binding between sorafenib and StkP-KD. Taken together, the computational simulations indicate a potential interaction of sorafenib with the catalytic site of pneumococcal StkP-KD.

### Sorafenib exhibits activity against multidrug-resistant pneumococci

**Fig 2:**
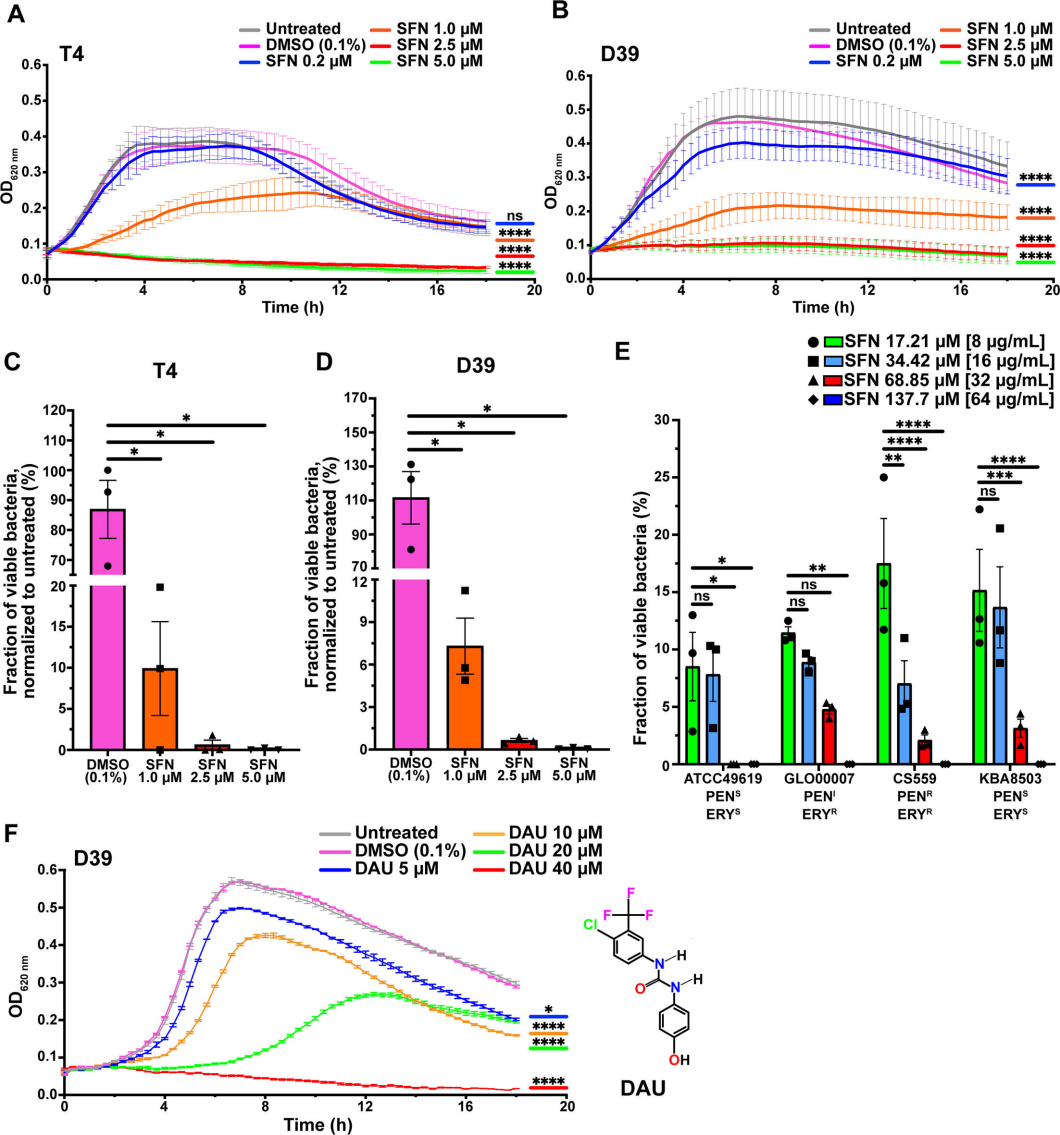
Sorafenib inhibits pneumococcal growth *in vitro*. (**A and B**). Growth kinetics of *S. pneumoniae* strains, (**A**) TIGR4 (T4) and (**B**) D39, showing dose-dependent inhibition of growth with increasing concentrations of sorafenib (SFN). DMSO-treated and untreated bacteria served as negative controls. **** indicates *P* ≤ 0.0001; ns denotes non-significance by the Mann-Whitney test relative to untreated. (**C–E**). Percentage viability of (**C**) T4, (**D**) D39, and (**E**) erythromycin (ERY) and penicillin (PEN) resistant and sensitive clinical strains of *S. pneumoniae* upon treatment with different concentrations of SFN. For clinical strains, R denotes resistant, S denotes sensitive, and I denotes intermediate following the CLSI criteria. * in panels **C **and **D** indicates *P* ≤ 0.05 relative to DMSO-treated bacteria by paired *t*-test. For panel **E**, * indicates *P* ≤ 0.05; ** indicates *P* ≤ 0.01; *** indicates *P* ≤ 0.001; **** indicates *P* ≤ 0.0001; ns denotes not significant by two-way ANOVA with Tukey’s multiple comparisons test. (**F**). Growth kinetics of D39 strain treated with diarylurea (DAU) showing higher MIC compared to SFN. Inset shows the structure of DAU. * indicates *P* ≤ 0.05, **** indicates *P* ≤ 0.0001 and ns denotes non-significance by the Mann-Whitney test relative to untreated. Data are representative of mean ± SEM from three independent experiments.

Next, we wanted to study the potential antimicrobial effects of sorafenib *in vitro*. Pneumococcal strains, TIGR4 (serotype 4; T4) and D39 (serotype 2), were grown in the presence of sorafenib at concentrations ranging between 0.2 and 5 μM. The equivalent DMSO concentration (0.1%) was used as the solvent control and maintained consistently across conditions. We found that sorafenib induced a dose-dependent inhibition of bacterial growth with a minimum inhibitory concentration (MIC) value of ~2.5 µM. (Fig. 2A and B). In agreement, CFU assays showed a dose-dependent reduction of 3–5 log-fold in comparison to the DMSO treatment, suggesting the bactericidal activity of sorafenib (Fig. 2C and D). To study whether capsular polysaccharide could influence the drug efficacy, we used the non-encapsulated strains T4R (serotype 4) and R6 (serotype 2) and found that they showed MIC values of 2.5 and 5 μM, respectively (Fig. S5A and B). Further, we tested the activity of sorafenib against six clinical pneumococcal strains, out of which five isolates were resistant to the commonly used macrolide, erythromycin, with a high MIC value of >128 µg/mL (Table 1). Our results showed that sorafenib was effective against all the strains tested, with an MIC value of 68.85 μM (32 μg/mL), irrespective of penicillin and erythromycin resistance. Interestingly, sorafenib showed ~4-fold lower MIC when compared to erythromycin in two clinical isolates, GLO00007 and SP676, with high erythromycin resistance and intermediate penicillin resistance (Table 1 and Fig. 2E). In agreement with the CFU assays, growth kinetic assays of clinical strains, serotypes 3 and 19F, showed complete growth inhibition at 17.21–137.7 μM suggesting bactericidal activity of sorafenib (Fig. S5C and D).

**TABLE 1:** MIC values of sorafenib against multidrug-resistant clinical strains of pneumococci^*a*^

ID	Child/adult	Source	Invasive/non-invasive	Diagnosis	Serotype	Penicillin MIC (μg/mL)	Erythromycin MIC (μg/mL)	Sorafenib MIC (μg/mL)[μM]	DMSO MIC (μg/mL)
GLO00007	Child	Pleural fluid	Invasive	Pneumonia	19A	4 (I)	>128 (R)	32 [68.85]	>128
SP676	Child	Sputum	Non-invasive	Respiratory tract infection	19F	4 (I)	>128 (R)	32 [68.85]	>128
SP2782	Adult	Sputum	Non-invasive	Respiratory tract infection	19F	2 (S)	>128 (R)	32 [68.85]	>128
CS559	Adult	CSF	Invasive	Meningitis	19F	2 (R)	8 (R)	32 [68.85]	>128
311104	Child	Nasopharyngeal swab	Non-invasive	Carriage	19F	2 (S)	8 (R)	32 [68.85]	>128
KBA8503	Adult	Blood	Invasive	Bacteremia	6C	0.12 (S)	0.25 (S)	32 [68.85]	>128

^*a*^ S, susceptible; I, intermediate; R, resistant following the CLSI criteria.

To dissect the significance of the pyridine moiety, a hallmark of kinase inhibition (29, 30) on the activity of sorafenib against pneumococcal growth inhibition, we synthesized the diarylurea (DAU) backbone of sorafenib (lacking pyridine moiety) along with the chloro, trifluoromethylphenyl, and hydroxyl functional groups (Fig. S5E), and the molecular weight and purity were validated by mass spectrometry and HPLC, respectively (Fig. S5F and G). Growth kinetic assays of D39 strain in the presence of DAU showed a ~16-fold increase in the MIC (~40 µM), compared to sorafenib (Fig. 2F), suggesting a potential role being contributed by the pyridine derivative, methylated picolinamide group of sorafenib. Therefore, next we were interested to see the effect of sorafenib on the kinase activity of pneumococcal StkP.

### Pneumococcal StkP is one of the potential targets of sorafenib

**Fig 3:**
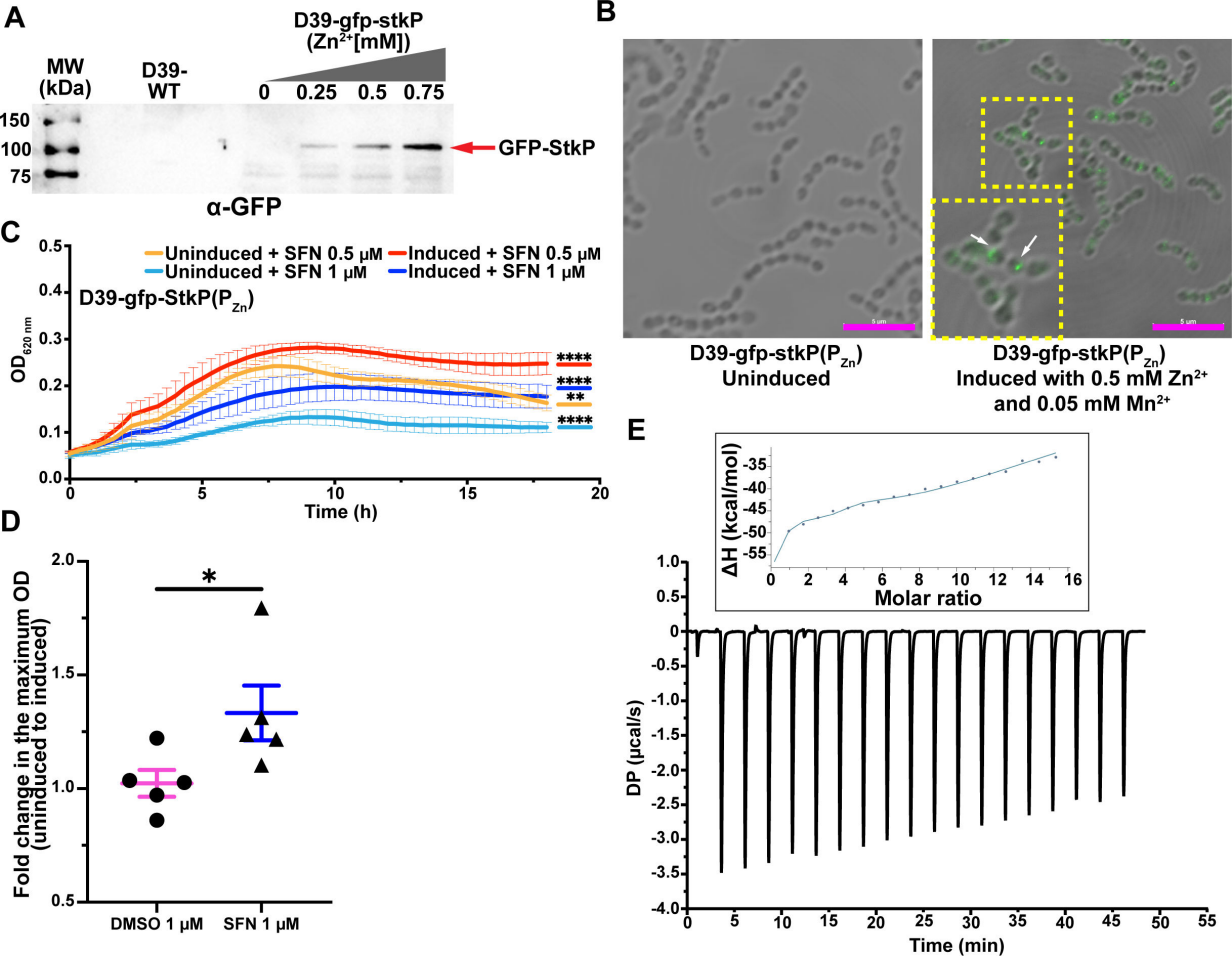
Pneumococcal StkP is one of the potential targets of sorafenib. (**A**) Western blot showing the dose-dependent increase in GFP-StkP expression levels with increase in Zn^2+^ concentration, probed with anti-GFP antibody. (**B**) Confocal microscopy shows the septal localization of GFP-StkP protein in D39-gfp-stkP(P_Zn_) strain upon induction with 0.5 mM ZnCl_2_ and 0.05 mM MnCl_2_. Inset shows the magnification of selected region. Scale bars, 5 µm. Images are representative of three independent experiments. (**C**) Growth kinetics of D39-gfp-stkP(P_Zn_) upon treatment with 0.5 and 1 µM of sorafenib (SFN) in both uninduced and induced (0.25 mM ZnCl_2_ and 0.025 mM MnCl_2_) conditions. ** indicates *P* ≤ 0.01 and **** indicates *P* ≤ 0.0001 comparing uninduced/induced SFN-treated bacteria to the respective untreated bacteria by the Mann-Whitney test. Data are representative of mean ± SEM from three independent experiments. (**D**) Fold change in the maximum OD_620_ of 1 µM DMSO or SFN-treated bacteria comparing induced to uninduced conditions. * indicates *P* ≤ 0.05 by paired *t*-test. Data are representative of mean ± SEM from five independent experiments. (**E**). Isothermal titration calorimetry thermogram showing heat changes from sequential injections of sorafenib (400 µM) into recombinant StkP-KD (5 µM), indicating exothermic binding. Inset shows the enthalpy (Δ*H*) per injection plotted as a function of the ligand-to-protein molar ratio. The data were fitted using a sequential five-site binding model to obtain the stepwise thermodynamic parameters.

To investigate whether StkP is a potential target of sorafenib, we used genetically engineered pneumococcal D39 strain expressing the N-terminal GFP-fused StkP (GFP-StkP) at the dispensable bgaA locus under a zinc-inducible P_czcD_ promoter (D39-gfp-stkP(P_Zn_)). The gradient induction of GFP-StkP in the D39-gfp-stkP(P_Zn_) strain using ZnCl_2_ was validated by western blotting (Fig. 3A). The septal localization of GFP-StkP in D39-gfp-stkP(P_Zn_) was confirmed by confocal microscopy (Fig. 3B) and flow cytometry (Fig. S6A) as previously reported (10). Growth kinetic assay showed that ectopic expression of StkP in D39-gfp-stkP(P_Zn_) strain was able to partly rescue growth in a dose-dependent manner to 0.5–1 µM of sorafenib (Fig. 3C). Upon StkP induction, D39-gfp-stkP(P_Zn_) showed a significant ~1.5-fold increase in growth compared to the uninduced condition in the presence of 1 µM sorafenib (Fig. 3D). In comparison, the control experiment with the solvent control, DMSO did not show a significant change compared to uninduced (Fig. S6B). Next, we recombinantly expressed and purified His-tagged StkP kinase domain (StkP-KD) in *Escherichia coli* BL21(DE3) cells (Fig. S6C) to study the interaction with sorafenib using isothermal titration calorimetry. Purified StkP in the sample cell was subjected to microcalorimetric titration with different molar ratios of sorafenib and the heat changes measured were subtracted with buffer alone control containing equivalent DMSO concentration. Binding kinetics showed a sharp reduction in differential power upon ligand injection indicative of exothermic reaction and subsequent gradual reduction approaching saturation (Fig. 3E). ITC titration data were analyzed using a five-site sequential binding model, revealing multiple thermodynamically distinct ligand-binding events. The fitted stepwise dissociation constants were determined to be *K_d_*_,1_ = 3.13 µM, *K_d_*_,2_ = 11.0 µM, *K_d_*_,3_ = 0.132 ± 9.84 µM, *K_d_*_,4_ = 111 ± 35.3 µM, and *K_d_*_,5_ = 136 ± 86.4 µM. These results suggest that ligand binding potentially occurs through five sequential equilibria with varying affinities.

### Sorafenib inhibits the kinase activity of StkP

**Fig 4:**
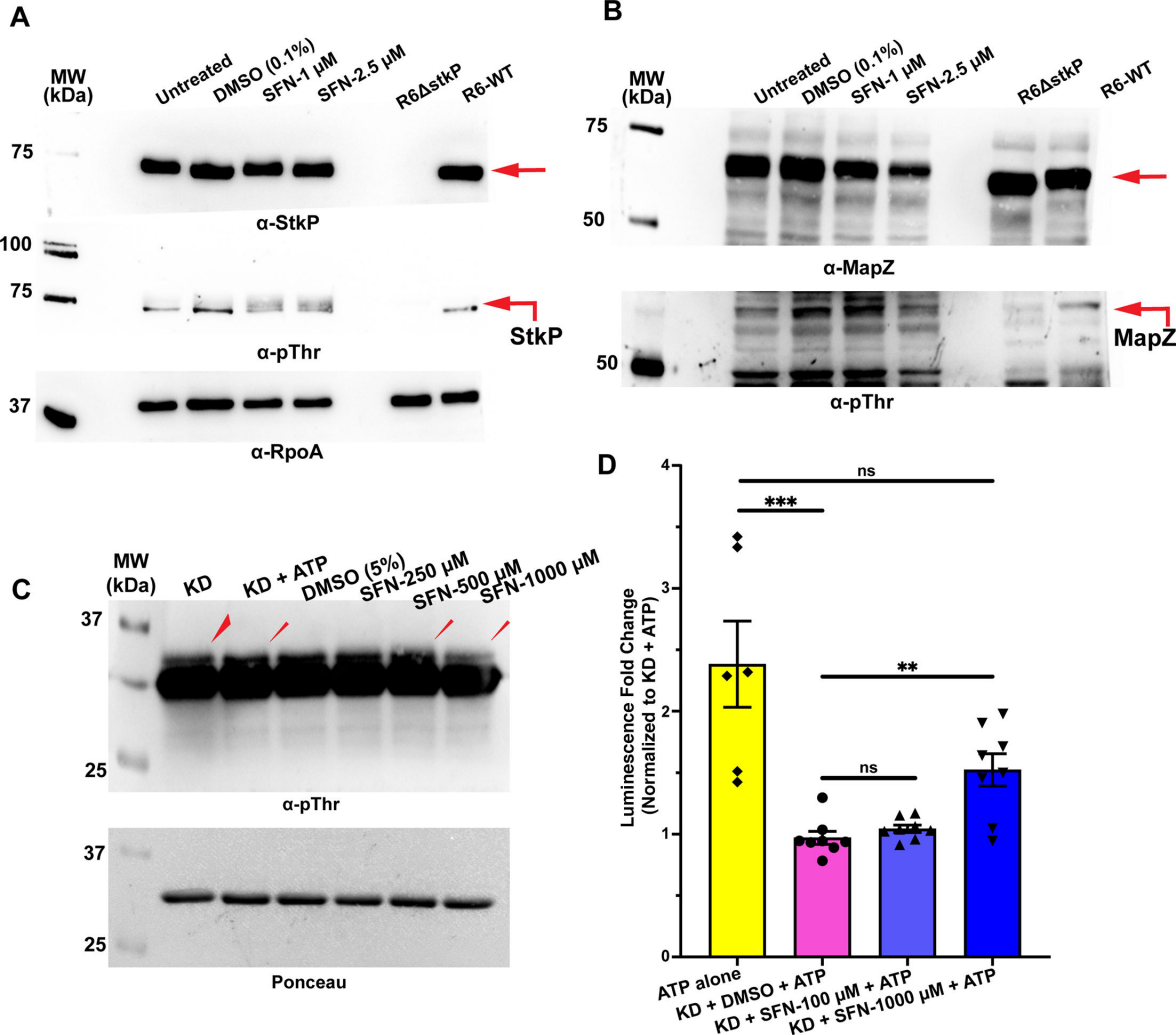
Sorafenib inhibits the kinase activity of StkP. (**A and B**). Western blots probed with p-Thr-specific antibody showing the downregulation of phosphorylation of (**A**) StkP and (**B**) MapZ (StkP substrate protein) in *S. pneumoniae* D39 strain upon treatment with 1 and 2.5 μM sorafenib (SFN). StkP and MapZ levels are shown as respective loading controls. Blots are representative of three independent experiments. (**C**) Western blots showing the *in vitro* autophosphorylation of 1 μM of purified recombinant StkP-KD treated with 250, 500, and 1,000 μM of SFN, in the presence of 250 µM of ATP. Equivalent concentration (5%) of DMSO treated and untreated KD in the presence of ATP were used as the controls. KD alone without any ATP serves as the basal level phosphorylation indicator. Blot is representative of four independent experiments. (**D**) Fold change in the luminescence signal of 100 and 1,000 μM SFN and equivalent DMSO (1%) treated StkP-KD in the presence of 10 μM of ATP, normalized to untreated KD in the presence of ATP. ATP alone without any protein served as the control. Data are representative of mean ± SEM from four independent experiments. ** indicates *P* ≤ 0.01; *** indicates *P* ≤ 0.001; and ns denotes non-significance by the Mann-Whitney test.

To investigate the effect of sorafenib on the autophosphorylation and kinase activity of StkP in *S. pneumoniae* D39 strain, we analyzed both the phosphorylation status of StkP as well as its substrate protein (MapZ) in treated cell lysates. Immunoblotting using a phospho-threonine-specific (anti-p-Thr) antibody revealed a reduction in the autophosphorylation levels of StkP upon treatment with sorafenib at 1 and 2.5 µM doses compared to DMSO-treated control (Fig. 4A). The total StkP levels were probed using anti-StkP antibody and served as the loading control. The unencapsulated strain, R6 and the isogenic StkP knockout strain, R6ΔstkP were used as additional controls to demonstrate the specificity of StkP and p-Thr antibodies. Unlike the D39 strain, StkP can be deleted in the R6 background as reported previously (31) due to a compensatory mutation in *murZ*(I265V) gene. In agreement, our data also showed no difference in growth between R6 and R6ΔstkP strains (Fig. S7A). The StkP substrate protein, MapZ showed a downregulation of both the total levels as well as its phosphorylated form (Fig. 4B). The RNA polymerase alpha subunit, RpoA, was used as a housekeeping control to show the specificity of StkP and MapZ regulation upon sorafenib treatment. Further, using mass-spectrometry, we identified other StkP substrate proteins that showed downregulation of phosphorylation, such as DivIVA and GpsB, which are involved in bacterial cell division (Table 2 and Fig. S7B).

**TABLE 2:** List of proteins in selected bands below 37 kDa in Fig. S7B identified from mass spectrometry

UNIPROT ID	Protein name	Mol wt (kDa)	Gene ontologies
A0A062WNB1	Cell division protein SepF	20.6	Division Septum Assembly [0000917], FtsZ-Dependent Cytokinesis [0043093]
A0A062WWL4	Glutamate racemase	29.1	Cell Wall Organization [0071555], Peptidoglycan Biosynthetic Process [0009252], Regulation of Cell Shape [0008360]
A0A064C1J4	Cell shape-determining protein MreC	29.7	Regulation of Cell Shape [0008360]
A0A098Z983	Phospho-N-acetylmuramoyl-pentapeptide-transferase	36	Cell Division [0051301], Cell Wall Organization [0071555], Peptidoglycan Biosynthetic Process [0009252], Regulation of Cell Shape [0008360]
A0A0B7LLG7	Undecaprenyl-diphosphatase	31.7	Cell Wall Organization [0071555], Peptidoglycan Biosynthetic Process [0009252], Regulation of Cell Shape [0008360]
A0A0H2ZM82	Cell division ATP-binding protein FtsE	25.8	Cell Division [0051301]
A0A0I7YM98	Cell cycle protein GpsB	13.1	Cell Division [0051301], Regulation of Cell Shape [0008360]
A0A4F3K9I6, B8ZLT0	Probable GTP-binding protein EngB	22.2	Division Septum Assembly [0000917]
A0A822Q745, A0A064C6I1	Cell division protein DivIVA	33.2	Cell Division [0051301]
A0A4J1QNE6	UDP-N-acetylenolpyruvoylglucosamine reductase	32.9	Cell Division [0051301], Cell Wall Organization [0071555], Peptidoglycan Biosynthetic Process [0009252], Regulation of Cell Shape [0008360]
Q4JZ00, Q4K2E2, Q4K046	Capsular polysaccharide biosynthesis protein CpsC	25.6	Capsular Polysaccharide Biosynthesis [0045227]
Q4JZ01	Tyrosine-protein phosphatase	28	Capsular Polysaccharide Biosynthesis [0045227]
Q4K2E2	Capsular polysaccharide biosynthesis protein CpsC	25.7	Capsular Polysaccharide Biosynthesis [0045227]
Q97QE5	Ribitol-5-phosphate cytidylyltransferase	26.2	Cell Wall Organization [0071555]
Q9ZII6	Tyrosine-protein kinase CpsD	24.8	Capsular Polysaccharide Biosynthesis [0045227]
A0A064C372	Probable manganese-dependent inorganic pyrophosphatase	33.5	
P66708	DNA-directed RNA polymerase subunit alpha	34.2	

Next, we performed *in vitro* kinase assay with purified recombinant StkP-KD to study its autophosphorylation status in the presence of sorafenib. StkP-KD exhibited a dose-dependent increase in the phosphorylated form with increasing ATP concentrations confirming the linearity of the assay (Fig. S7C). Notably, incubation with sorafenib, but not with equivalent concentrations of the solvent DMSO, led to a dose-dependent reduction in StkP-KD phosphorylation (Fig. 4C), suggesting an inhibitory effect of sorafenib on StkP autophosphorylation activity. The total protein stained with Ponceau served as the loading control. Since the purified StkP-KD was already showing a basal level of phosphorylation upon expression in *E. coli*, we directly examined the autophosphorylation of StkP-KD in transformed *E. coli* cultures grown without IPTG induction to permit only basal-level StkP expression and grown in the presence of sorafenib. Our results demonstrated that sorafenib caused a significant and dose-dependent reduction of StkP phosphorylation in *E. coli*, relative to untreated and DMSO controls (Fig. S7D). The phosphorylated band was quantified by densitometry analysis (Fig. S7E). The total *E. coli* lysate proteins stained with Coomassie were used as the loading control (Fig. S7F). Given that *E. coli* lacks eukaryotic-like Ser/Thr kinases and is therefore not inherently involved in the regulation of cell division (32), we hypothesized that sorafenib would not affect bacterial growth in this heterologous expression system. Consistent with this, the growth kinetics of *E. coli* expressing StkP-KD remained unaltered in the presence of sorafenib, supporting the specificity of its inhibitory effect (Fig. S7G).

To precisely quantify the autophosphorylation activity of the purified StkP-KD, we used the Kinase-Glo luciferase assay (Promega) that correlates with the amount of ATP left after kinase reaction and is inversely proportional to the kinase activity. The dose-dependent response of the kinase assay to free ATP upon titration of StkP-KD with ATP gradient was established (Fig. S7H). Compared to ATP alone, StkP-KD addition showed a reduction of signal indicating ATP binding (Fig. 4D). In agreement with *in vitro* autophosphorylation assay (Fig. 4C), a dose-dependent reduction in kinase activity was observed upon incubating the protein with sorafenib, confirming the direct inhibitory effect of sorafenib on StkP autophosphorylation activity (Fig. 4D). Together, the above results suggest an inhibitory effect of sorafenib on the StkP kinase activity.

### Sorafenib counters bacterial fitness leading to complement deposition and serum killing

**Fig 5:**
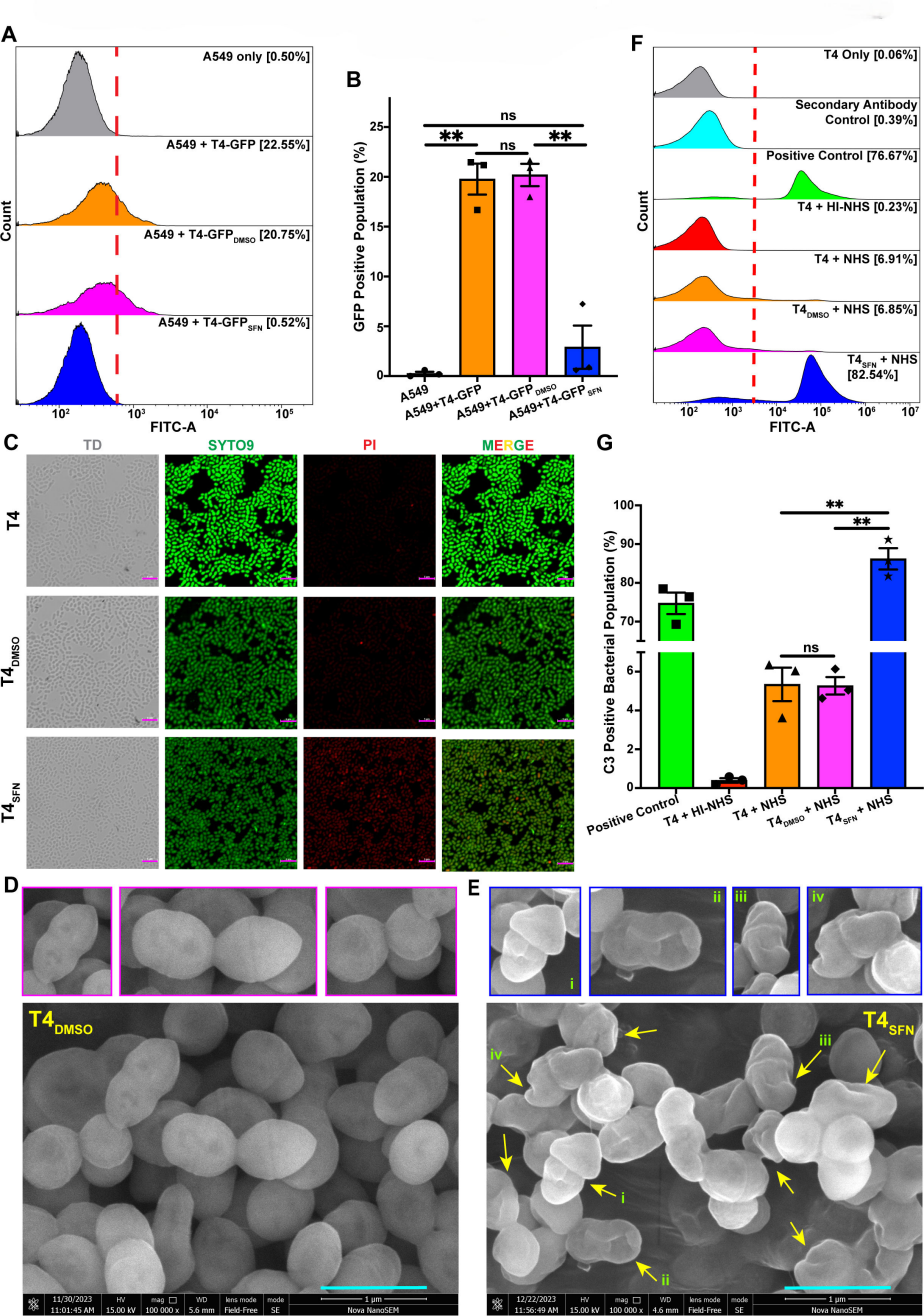
Sorafenib counters bacterial fitness leading to complement deposition and serum killing. (**A**) Flow cytometry histograms of human A549 epithelial cells infected with GFP-expressing *S. pneumoniae* TIGR4 strain (T4-GFP) grown in the presence or absence of 10 μM sorafenib (SFN). Treatment with equimolar concentrations of DMSO served as control. (**B**) Quantification of the percentage of GFP-positive infected cells in panel **A** showing reduced infection of A549 cells by SFN-treated bacteria. ** indicates *P* ≤ 0.01; ns denotes non-significance by the Welch’s *t*-test. (**C**) Viability staining of TIGR4 strain treated with 10 μM SFN using SYTO9 and PI dyes showing differential staining of live (green) and dead (red and green) bacteria. Scale bars, 5 µm. (**D and E**) Scanning electron microscopy of (**D**) 10 μM DMSO (solvent) and (**E**) SFN-treated bacteria showing aberrant cell morphology. Inset shows the magnification of regions selected with compromised cell wall. Magnification 100,000×. Scale bars, 1 µm. (**F**) Flow cytometry histogram and (**G**) quantification analysis of complement C3 protein deposition on 10 μM SFN-treated T4 bacteria upon incubation with normal healthy serum (NHS). Heat inactivated serum (HI-NHS) was used as negative control. Capsule permeabilized bacteria that were opsonized with anti-*S. pneumoniae* antibody was used as the positive control. ** indicates *P* ≤ 0.01 and ns denotes non-significance by the paired normal *t*-test. All data and images in panels A–G are representative of mean ± SEM from three independent experiments.

We next studied the impact of sorafenib treatment on key bacterial virulence-associated phenotypes, including host cell invasion, membrane integrity, morphological alterations, and susceptibility to complement-mediated killing. We used the GFP-expressing *S. pneumoniae* TIGR4 reporter strain (T4-GFP) to assess bacterial invasion into human A549 lung epithelial cells. A549 cells were infected with T4-GFP pre-treated with 10 µM sorafenib for 3 h, and the proportion of GFP-positive host cells was quantified by flow cytometry. The selected concentration of sorafenib reflects the clinically achievable plasma levels in patients undergoing cancer therapy (33). While infection with untreated or DMSO-treated bacteria resulted in a positive peak shift in the cell-associated bacterial GFP signal, sorafenib-treated bacteria showed reduced GFP signal comparable to uninfected controls (Fig. 5A) with a significant reduction in the percentage of infected cells (Fig. 5B). To measure host cytotoxicity at the effective bactericidal concentrations, we performed PrestoBlue cell viability assay on A549 cells treated with sorafenib or DMSO across a range of concentrations (2.5–10 µM). No significant cytotoxicity was observed (Fig. S8A). Cells treated with 5 µM staurosporine, a known inducer of apoptosis, showed a ~50% reduction in viability and served as the positive control for cytotoxicity. Next, we examined whether sorafenib affects bacterial membrane permeability. Differential viability staining using SYTO9 (green), which stains all bacteria, and propidium iodide (PI, red), which penetrates only cells with compromised membranes, revealed increased double-positive stained population upon sorafenib treatment (Fig. 5C). In contrast, untreated and DMSO-treated controls showed negligible PI staining. These results suggest that sorafenib induces significant membrane damage, which was further confirmed by flow cytometric analysis showing a significantly higher proportion of PI-positive cells in the sorafenib-treated group (Fig. S8B). To visualize the effects of sorafenib on bacterial morphology, we performed scanning electron microscopy analysis of T4 bacteria grown in the presence of 10 μM sorafenib. Bacteria treated with the solvent alone, DMSO, had normal morphology (Fig. 5D), while sorafenib-treated bacteria exhibited abnormal morphology with surface deformations indicative of compromised cell wall (Fig. 5E). Given the aberrant morphology and membrane disruption, we next assessed the susceptibility of sorafenib-treated bacteria to complement-mediated opsonization. T4 bacteria treated with sorafenib, DMSO, or left untreated were incubated with human serum from healthy donors, and complement protein C3 bound to the bacterial membrane was quantified by flow cytometry. Sorafenib-treated bacteria showed significantly increased C3 deposition when compared to DMSO or untreated groups (Fig. 5F and G), both of which exhibited minimal complement binding due to the protective polysaccharide capsule. Notably, the level of C3 deposition on sorafenib-treated bacteria was comparable to capsule-permeabilized bacteria opsonized with anti*-S. pneumoniae* antibody, which served as the positive control. Bacteria incubated with heat-inactivated serum served as the negative control and showed negligible C3 binding.

To test whether the enhanced C3 deposition observed upon sorafenib treatment translated into higher bacterial killing, bacterial CFUs were determined upon incubation with human serum. As expected, untreated and DMSO-treated bacteria incubated with serum showed similar CFU counts regardless of serum exposure, indicating negligible serum-mediated killing under these conditions. In contrast, sorafenib-treated bacteria showed a significant reduction in CFU counts both in the presence and absence of serum, consistent with the direct bactericidal activity of sorafenib (Fig. S8C). Collectively, these findings demonstrate that sorafenib inhibits key bacterial virulence traits, including host cell invasion and immune evasion, by inducing membrane disruption and morphological defects, ultimately rendering *S. pneumoniae* highly susceptible to complement-mediated opsonization and killing.

### Limited potential for resistance emergence upon serial passaging with sorafenib

**Fig 6:**
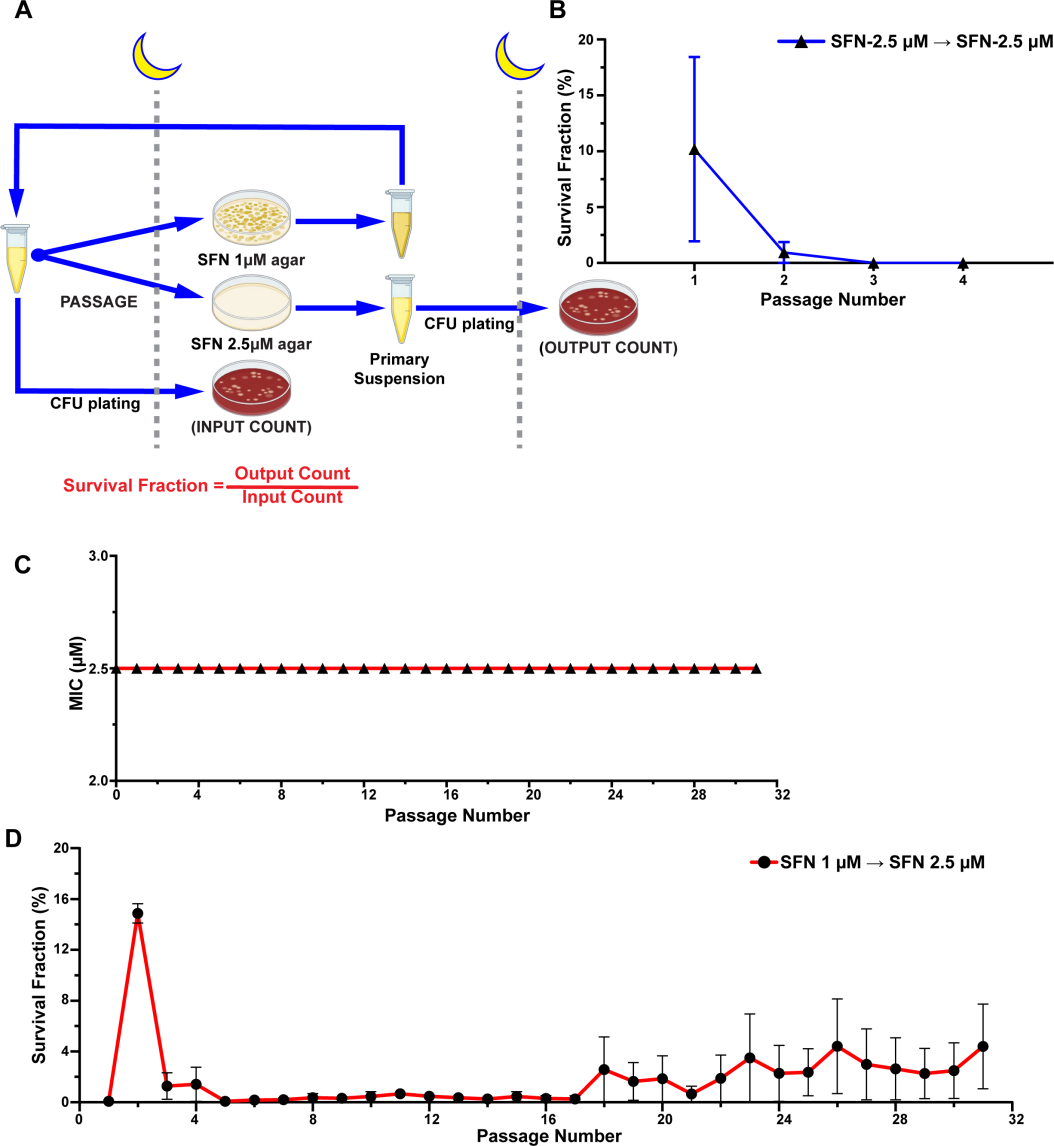
Limited potential for resistance emergence upon serial passaging with sorafenib. (**A**) Schematic showing the strategy of serial passaging of T4 strain with sub-MIC (1 µM) and MIC (2.5 µM) doses of sorafenib (SFN), followed by CFU enumeration of the respective plates. The passaging was performed 4× under 2.5 µM dose after which no CFU was recovered and 31× in 1 µM dose (**B**). Bacterial survival fraction at each passage upon serial passaging of T4 on BHI agar plates containing 2.5 μM (MIC) of SFN showing gradual decline up to passage 3 after which no CFU was recovered. The ratio of output CFU recovered at the end of passage to the input from the previous passage was used to calculate the survival fraction. Data are representative of three independent experiments. (**C**) Change in MIC upon serial passaging at 1 μM SFN and plating in BHI agar supplemented with 2.5 μM SFN. (**D**) Bacterial survival fraction showing viable pneumococci recovered on 2.5 μM SFN plates post successive passages with 1 μM SFN. Data in panels **C** and **D** are representative of mean ± SEM from two independent experiments.

To evaluate the potential for resistance development against sorafenib, we performed serial passaging of *S. pneumoniae* T4 strain in the presence of the drug. Bacteria were sequentially cultured on agar plates containing either the MIC concentration (2.5 µM) and sub-MIC concentration (1 µM) of sorafenib, and CFUs were quantified at each passage using plating assays (Fig. 6A). Following three consecutive passages at 2.5 µM, bacterial CFUs declined below the limit of detection, indicating an inability to propagate under continued drug pressure (Fig. 6B). Due to growth limitation at the MIC concentration, extended serial passaging was carried out at the sub-MIC level (1 µM). Throughout the experiment, no change in the MIC value was observed (Fig. 6C). Furthermore, analysis of CFU recovery at each passage revealed that the output CFUs remained consistently low, never exceeding 10–15% of the input CFUs by the end of passage 31 with no change in the MIC value (Fig. 6D). Collectively, these results suggest that under the tested conditions, no significant drug resistance to sorafenib was observed in *S. pneumoniae*.

### Sorafenib treatment delays disease progression and bacterial load *in vivo*

**Fig 7:**
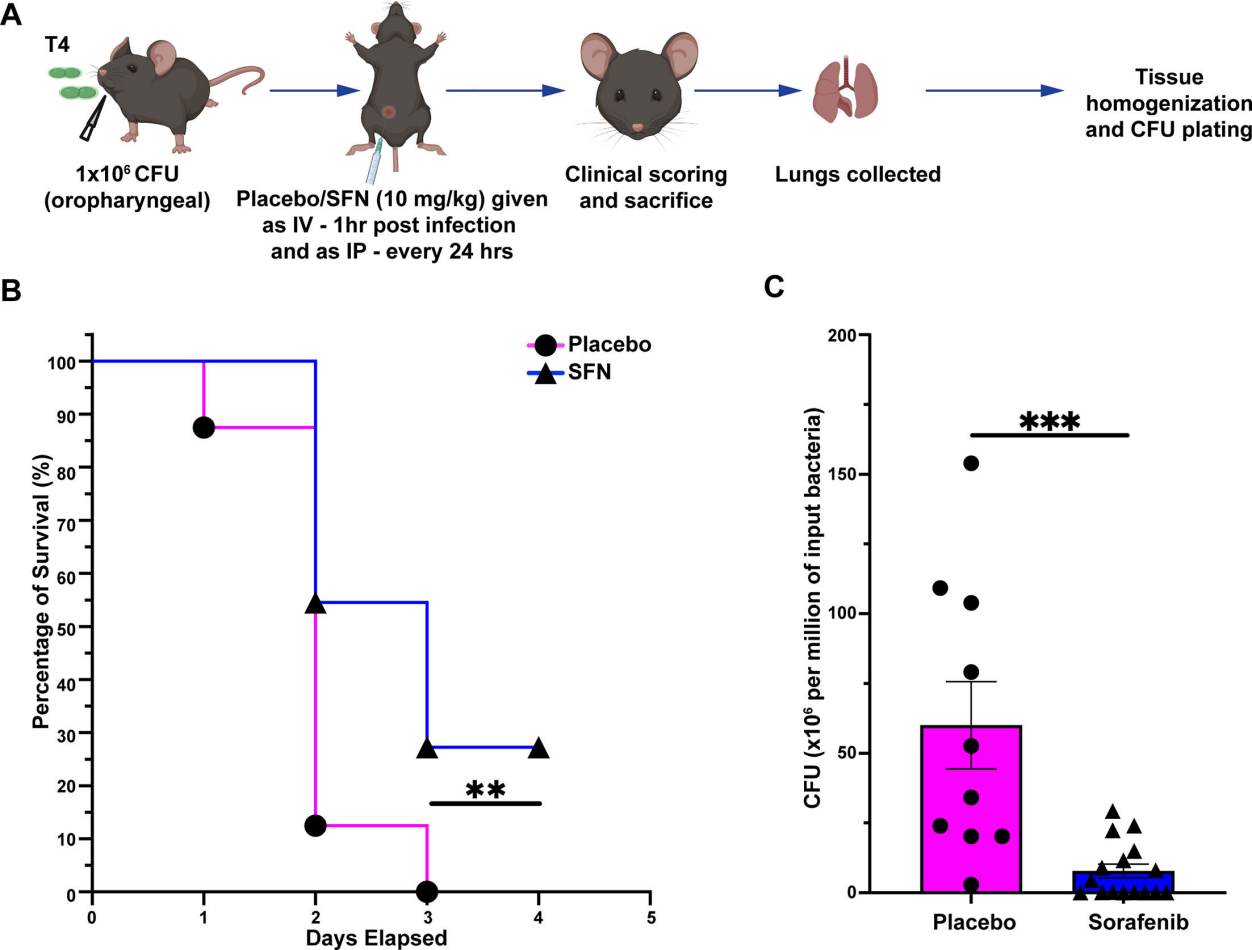
Sorafenib treatment delays pneumococcal disease progression and bacterial load *in vivo*. (**A**) Schematic showing the experimental workflow for testing the efficacy of sorafenib in 6- to 9-week-old male C57BL/6 mice infected with 1 × 10^6^ CFU of TIGR4 strain via oropharyngeal route, followed by treatment with sorafenib (SFN) (10 mg/kg) intravenously at 1 h post-infection and intraperitoneally at 24 h intervals. Infected mice treated with the solvent, 55% PEG-400 and 20% DMSO, were used as the placebo control. Created with BioRender.com. (**B**) Survival curve of C57BL/6 mice (*n* = 16 for placebo; *n* = 11 for SFN) infected with T4 strain and treated with SFN or placebo over 4 days post-infection. Infected mice were scored for clinical symptoms daily and were sacrificed upon reaching the ethical end point. ** indicates *P* ≤ 0.01 by the Mantel-Cox test. (**C**) Bacterial CFU (in millions) recovered from the lungs of infected mice (*n* = 10 for placebo; *n* = 16 for SFN) was measured post‐sacrifice and expressed normalized per million of input bacteria. *** indicates *P* ≤ 0.001 by the Mann-Whitney test. Data in panels **B** and **C** are representative of mean ± SEM.

We next evaluated the *in vivo* efficacy of sorafenib in a murine model of pneumococcal pneumonia. Pneumonia was induced in 6- to 9-week-old male C57BL/6 mice via oropharyngeal administration of 1 × 10^6^ CFU of the *S. pneumoniae* serotype 4 strain, TIGR4. Mice in the treatment group received an initial intravenous dose of sorafenib (10 mg/kg in 50 µL of 55% PEG-400 and 20% DMSO) at 1 h post-infection, followed by intraperitoneal doses at 24 h intervals until the ethical endpoint was reached (Fig. 7A). The 10 mg/kg dose was selected to mimic a clinically relevant low-dose sorafenib regimen (equivalent to ~45  mg/kg in humans), which represents ~10% of the dose typically used for hepatocellular carcinoma treatment (34, 35), and is approximately one-third of the reported maximum tolerated dose in mice (36). Placebo mice received an equivalent volume of the vehicle (55% PEG-400 and 20% DMSO). Sorafenib-treated mice showed delayed disease progression and exhibited a ~30% survival compared to placebo-treated controls by day 4 post-infection, whereas all mice in the placebo group succumbed by day 3 (Fig. 7B). Consistent with greater survival, lung homogenates from sorafenib-treated mice showed ~10-fold reduction in bacterial burden relative to control (Fig. 7C). Collectively, these results demonstrate that sorafenib treatment reduces pulmonary bacterial load and improves survival outcomes in a mouse model of pneumococcal pneumonia.

## DISCUSSION

The growing threat of antimicrobial resistance in *S. pneumoniae* underscores the urgent need for novel therapeutic strategies. In this context, repurposing FDA-approved drugs with antimicrobial activity offers a promising alternative strategy by passing *de novo* drug design and pharmacological optimization. Our study demonstrates the repurposing potential of sorafenib, a clinically approved multi-kinase inhibitor currently used in the treatment of thyroid, renal, and hepatocellular carcinomas (37), for treating pneumococcal infections and identifies the serine threonine kinase, StkP, as one of the potential targets.

We identified sorafenib through *in silico* screening of host-directed kinase inhibitors against the StkP kinase domain. Molecular dynamics simulations indicated possible interaction of sorafenib within the catalytic cleft of StkP, in competition with its natural substrate, DivIVA. These findings provided a basis for experimental validation of StkP inhibition by sorafenib. We also demonstrated direct inhibition of StkP autophosphorylation activity through *in vitro* kinase assay with purified kinase domain as well as p-Thr blotting in the *E. coli* expression system. The StkP kinase domain used in our study contains two conserved threonine autophosphorylation sites within the activation loop (38) but displays lower autophosphorylation relative to the full-length protein, owing to the absence of the juxtamembrane domain, which harbors seven additional sites (39).

In agreement with *in vitro* kinase assay, phospho-threonine profiling of StkP and its substrate protein, MapZ, in sorafenib-treated pneumococci revealed a reduction in phosphorylation levels. Besides, mass-spectrometry identification of protein bands showing reduced phosphorylation in treated bacteria revealed many other proteins involved in cell wall organization, division, and capsular biosynthesis such as DivIVA, GpsB, MreC, Undecaprenyl-diphosphatase, CpsC, and CpsD. Genetic rescue experiments further suggest StkP as a functional target, as ectopic expression of StkP restored growth in the presence of sorafenib. Isothermal titration calorimetry showed direct binding of sorafenib to StkP-kinase domain, indicating sequential binding model with multiple thermodynamically distinct ligand-binding events.

Sorafenib-treated bacteria exhibited morphological abnormalities and compromised membrane integrity, phenocopying StkP effector mutants, such as DivIVA, GpsB, and MapZ (17). Identification of altered phosphorylation of StkP substrate protein, MapZ may support growth defects as MapZ functions as a spatial landmark that guides FtsZ ring positioning and septum placement during the pneumococcal cell cycle. Given the role of biosynthesis proteins CpsC and CpsD in regulating capsule synthesis and its integration with cell cycle control, perturbation of these proteins may likely contribute to enhanced C3 deposition, increased serum susceptibility, and reduced epithelial adherence and invasion. In addition, impaired adherence and invasion of alveolar epithelial cells of sorafenib-treated bacteria are consistent with the established role of StkP in coordinating surface-associated virulence determinants (12, 40).

Importantly, although our data support StkP as a potential functionally relevant target of sorafenib in *S. pneumoniae*, we acknowledge that this does not exclude other targets such as potentially in cell wall or energy metabolism pathways. Sorafenib is a diarylurea compound with known polypharmacology, and related scaffolds have been shown to engage non-kinase bacterial targets. Notably (16), identified demethylmenaquinone methyltransferase (MenG), a key enzyme in menaquinone biosynthesis, as the primary bacterial target of a sorafenib-derived compound in MRSA, demonstrating kinase-independent antibacterial activity linked to disruption of electron transport. Consistent with this, prior reports of sorafenib activity against MRSA did not implicate a bacterial kinase. Our experiments with the diarylurea (DAU) backbone of sorafenib supported the role of the kinase inhibitory pyridine moiety towards the observed phenotypes.

**Fig 8:**
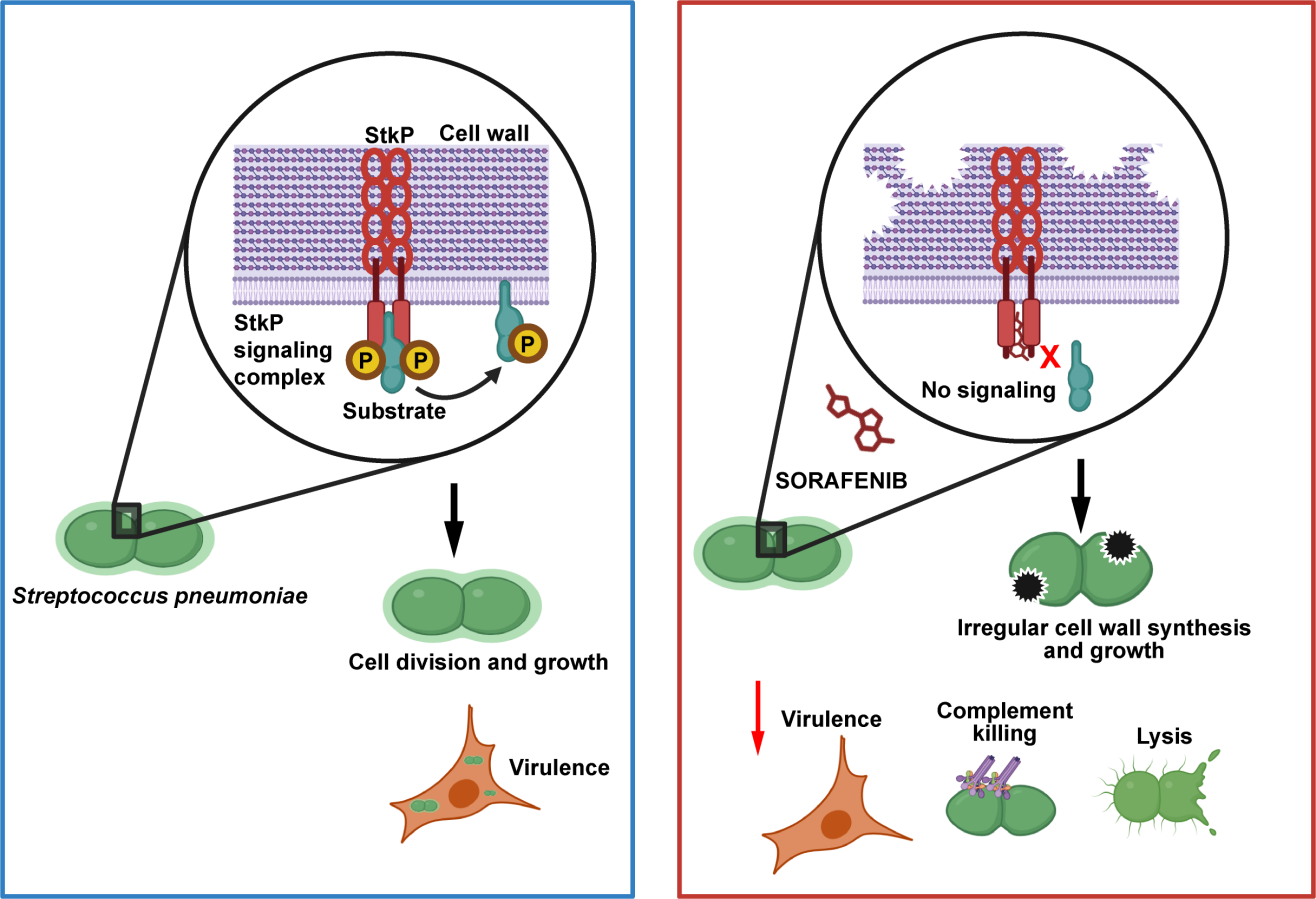
Sorafenib counters pneumococcal growth and virulence through StkP as one of the potential targets. Schematic summarizing the antimicrobial effects of sorafenib against *S. pneumoniae*. The serine threonine kinase, StkP plays a vital role in bacterial growth by coordinating cell wall synthesis and septal division. Our study suggests that sorafenib inhibits StkP kinase activity potentially through interaction with the catalytic cleft, resulting in irregular cell wall and blockade of growth. Sorafenib-treated bacteria showed reduced bacterial invasion into lung epithelial cells and enhanced membrane permeability and complement-mediated killing in serum. The *in vivo* efficacy was also validated in a murine model of pneumonia showing delayed disease progression and reduced bacterial load. Created with BioRender.com.

Sorafenib exhibited dose-dependent antimicrobial activity against laboratory and multidrug-resistant clinical isolates, including strains with high-level erythromycin resistance, and impaired key virulence traits at concentrations (2.5–10 µM) that were minimally cytotoxic to host cells. These concentrations fall within plasma levels of ~12 µM in pediatric patients with solid tumors and refractory leukemias, administered with 200 mg/m^2^ dose (41) and up to 20 µM in adults receiving a 400 mg dose (42). In a murine pneumonia model, sorafenib delayed disease progression and reduced pulmonary bacterial burden at a dose substantially below those used in cancer therapy, although larger and longer-term studies will be required to define optimal dosing and safety in infectious contexts. In conclusion, this study provides evidence that sorafenib impairs key virulence and survival mechanisms in *S. pneumoniae* via inhibition of the conserved bacterial kinase StkP as one of the potential mechanisms (Fig. 8). Targeting bacterial Ser/Thr kinases may represent a promising strategy to combat antimicrobial resistance. Further development and optimization of sorafenib and related kinase inhibitors targeting StkP could yield a new class of antimicrobials against pneumococci and other high-priority pathogens.

## MATERIALS AND METHODS

### Protein structure prediction and assessment

The protein sequence data of *S. pneumoniae* (strain ATCC BAA-334/TIGR4) StkP was obtained from the UNIPROT database (43) under the accession code Q97PA9. Due to the nonavailability of crystal structure for the pneumococcal StkP, particularly its kinase domain spanning residues 12–273, we employed AlphaFold (44) to generate a structural model for this domain. Specifically, we trimmed the structure for the residues 1–299 (StkP-KD), which was further processed using the Protein Preparation Wizard of Schrödinger, LLC, for refining the protein model. Finally, we utilized the structure assessment tool within the SWISS-MODEL server (45) to generate and analyze the Ramachandran plot of the constructed model, which helps to evaluate the stereochemical quality and stability of the protein model.

Furthermore, we have used PyMOL (PyMOL Molecular Graphics System, Version 3.0 Schrödinger, LLC) for structural superimposition and visualization of the alignments and to calculate the root-mean-square deviation (RMSD) between the constructed model of StkP-KD and the corresponding structures of *S. aureus* serine/threonine kinase protein—PknB (PDB ID: 4EQM) and *M. tuberculosis* serine/threonine kinase proteins, PknB (PDB ID: 3ORI) and PknA (PDB ID: 4OW8) to quantify structural similarities and deviations. Sequence alignment diagram was made using the ESPript 3.0 (ENDscript: https://endscript.ibcp.fr) (46).

### Virtual screening of ligands and docking with StkP

The prepared ligands were docked into the protein at the generated grid site using the extra precision (XP) docking algorithm in the Glide module (27) (Schrödinger Release 2024-4: Glide; Schrödinger, LLC, New York, NY) of Schrödinger. Ligands were sampled flexibly, with nitrogen inversions and ring conformations considered, while macrocycles were not sampled, and the input ring conformation was excluded. Torsion sampling was biased for all predefined functional groups, and Epik state penalties were added to the docking score. No core, shape, or torsional constraints were applied during docking. Post-docking minimization was performed, limiting the number of poses per ligand to 5. The output was saved as a pose viewer file, including the protein structure.

### Molecular dynamics (MD) simulations

The docked structure of the StkP-KD and sorafenib complex was loaded into Maestro and prepared using the Protein Preparation Wizard, as described previously for docking preparations. Molecular dynamics (MD) simulations were conducted using the Schrödinger Desmond software v.2023-2 (47) (Schrödinger Release 2024-4; Desmond Molecular Dynamics System, New York, NY) for a duration of 200 ns, with frames recorded every 100 ps. An orthorhombic box was created around the prepared StkP-KD-sorafenib complex with a 10 Å buffer of solvent molecules using the System Builder module. The box volume was minimized, and ions were added to neutralize the system. TIP3P solvent molecules were used for solvation (48). The system’s temperature was maintained at 300 K using the Nose-Hoover chain thermostat (49) with a relaxation time of 1.0 ps, while the pressure was kept at 1.01 bar using the Martyna-Tobias-Klein barostat (50) with isotropic coupling and a relaxation time of 2.0 ps. Coulombic interactions were handled using a short-range cutoff method with a cutoff distance of 9.0 Å. The OPLS4 force field was used to describe the interactions within the system.

DivIVA is a widely phosphorylated natural substrate of pneumococcal StkP (18). The full-length protein is 262 amino acids and Thr-201 is an important residue that is phosphorylated by StkP (UNIPROT ID: Q8CWP9) (28). Since the crystal structure of DivIVA is not available, we processed the AlphaFold predicted structure of DivIVA to extract a nine-amino-acid-containing peptide centered at Thr-201. The peptide was added near the catalytic cleft of StkP-KD complexed with sorafenib and prepared using the Protein Preparation Wizard. The interactions were simulated for 200 ns as previously described.

### Prime MM/GBSA calculations

The binding affinity of the ligand from MD simulation trajectories was calculated using the molecular mechanics-generalized born surface area (MM/GBSA) method implemented in the Prime module of Schrödinger Suite (51). The out.cms file from the MD simulation, which contains the trajectory data, was used as the input along with the specified ligand residue number. The binding free energy, which measures the thermodynamic stability of the protein-ligand interaction, was calculated using the following equation:


$$\Delta G_{\left\{bind\right\}}=\;G_{\left\{complex\right\}}-\;\left(G_{\left\{protein\right\}}+\;G_{\left\{ligand\right\}}\right)$$

Here, *G*_complex_ is the free energy of the protein-ligand complex, *G*_protein_ is the free energy of the unbound protein, and *G*_ligand_ is the free energy of the unbound ligand.

### Bacterial strains and culture

The encapsulated strains of *S. pneumoniae* serotype 4, TIGR4 (T4; ATCC BAA-334) and serotype 2, D39 (NCTC 7466) were kindly gifted by Prof. Birgitta Henriques Normark, Karolinska Institutet, Stockholm. The GFP-expressing T4 strain, T4-GFP, was a kind gift from Prof. Anirban Banerjee, Indian Institute of Technology, Bombay. The D39-gfp-stkP(P_czcD_, referred to as P_Zn_) strain with an inducible copy of stkP inserted at the dispensable beta-galactosidase (bgaA) locus and the R6ΔstkP strains were created using pJWV25-stkP (10) and AkCmDk plasmids (52) (kindly gifted by Dr. Pavel Branny, Czech Academy of Sciences, Czech Republic) as described below.

The TIGR4, D39, and R6 strains were streaked from frozen glycerol stocks on soybean casein digest agar plates supplemented with 5% sheep blood (HiMedia) and grown at 37°C and 5% CO_2_ overnight. T4-GFP and R6ΔstkP strains were grown on BHI agar (HiMedia) supplemented with 4 and 4.5 μg/mL of chloramphenicol (Sigma-Aldrich), respectively. D39-gfp-stkP(P_Zn_) was streaked on a BHI agar plate containing 2.5 μg/mL of tetracycline (HiMedia). To induce the gfp-stkP gene under the P_Zn_ promoter, the strain was streaked on a BHI agar plate containing 2.5 μg/mL of tetracycline, 0.5 mM of Zn^2+^ (as ZnCl_2_) (HiMedia), and 0.05 mM of Mn^2+^ [as Mn(II)Cl_2_] (HiMedia). All strains were grown in brain-heart infusion (BHI) broth (HiMedia) for suspension cultures with appropriate antibiotics and inducing reagents. *E. coli* strains were grown on Luria-Bertani (LB) broth and agar with 100 μg/mL of ampicillin.

For all inhibitor-based assays, sorafenib (Sigma-Aldrich) was dissolved in DMSO (HiMedia) to make 100 mM stocks. This stock was further diluted in DMSO to make 1,000× of experimental concentrations. These sub-stocks were directly added to the pneumococcal cultures, effectively making the final DMSO concentration 0.1% in all sorafenib-treated samples. For experiments involving *E. coli* and purified proteins, 100 mM sorafenib stock was diluted in DMSO to make 100× sub-stocks and used directly, which made the final concentration of DMSO 1% in all samples unless specified. 0.1% or 1% DMSO-treated cultures/samples were used as the solvent control for respective experiments.

### Generation and validation of gfp-stkP overexpressing and knockout strains

*S. pneumoniae* D39 strain was induced to competence by stimulation with 10 µg/mL of CSP-I (AnaSpec) in BHI competence media (10 mL BHI broth, 100 μL of 100 mM CaCl_2_, and 250 μL of 8% BSA; pH 8.0) for 10 min at 30°C. The competent cells were transformed with 1 μg of pJWV25-stkP plasmid to create D39-gfp-stkP(P_Zn_). The mixture was then incubated for 20 min at 30°C and subsequently for 2 h at 37°C, followed by plating on BHI agar containing tetracycline at 2.5 μg/mL concentration. The gfp-stkP gene was induced in BHI broth cultures using 0.5 mM Zn^2+^ (as ZnCl_2_) and 0.05 mM Mn^2+^ ]as Mn(II)Cl_2_] as reported previously (10, 53). GFP-StkP expression was validated using the CytoFlex S Flow Cytometer (Beckman Coulter). The data were analyzed using Kaluza version 2.2.1. The protein localization was validated by using a Nikon Eclipse Ti2 Inverted Confocal Imaging System. D39-WT and D39-gfp-stkP(P_Zn_) grown in the absence of Zn^2+^ and Mn^2+^ were used as negative controls.

The protein expression was also confirmed by western blotting. The bacterial pellets were lysed using RIPA lysis buffer (HiMedia) containing protease inhibitor cocktail (Roche) on ice for 30 min. Thirty microliters of the total lysate of D39-WT and D39-gfp-stkP(P_Zn_) were resolved on a 12% SDS-PAGE, and the proteins were transferred to a PVDF membrane using the Transblot Turbo Transfer System (Bio-Rad) at 1.3 A and 25 V for 15 min. The membrane was blocked using 5% BSA (HiMedia) in TBS buffer for 1 h at room temperature and then incubated with goat-induced anti-GFP antibody (Santa Cruz) at a 1:400 dilution overnight at 4°C. Post washes with TBS buffer with 0.1% Tween-20 (Bio-Rad) (0.1% TBST), the membrane was then probed using HRP-conjugated anti-goat secondary antibody (Bio-Rad) at 1:2,500 dilution and StrepTactin-HRP Conjugated secondary (Bio-Rad) at 1:10,000 dilution to detect Strep-tagged Precision Plus Protein WesternC Blotting Standards (Bio-Rad) for 1 h at room temperature. Post two washes with 0.1% TBST, the membrane was developed using Clarity Western ECL Substrates (Bio-Rad), and the luminescence was acquired using iBright FL1500 Imaging System (Invitrogen).

The R6ΔstkP strain was generated by transforming the R6 strain with 1 μg of AkCmDk plasmid post competence induction and was plated on BHI agar containing 4.5 µg/mL of chloramphenicol. Positive clones were confirmed by western blotting for StkP.

### Cell culture

Human adenocarcinoma alveolar basal epithelial cells, A549 (ATCC CCL-185), were procured from the National Centre for Cell Sciences, Pune, India. The cell lines were authenticated using a short tandem repeat 10 analysis and tested negative for mycoplasma contamination. A549 cells were grown in Dulbecco’s modified Eagle medium (DMEM) media (HiMedia) supplemented by 10% fetal bovine serum (FBS) (GIBCO) and 1% penicillin-streptomycin antibiotic (HiMedia) and were incubated at 37°C and 5% CO_2_.

### Bacterial growth kinetics assay

To measure the effect of sorafenib on pneumococci, growth kinetic assays were performed using the MultiSkan FC Microplate reader (ThermoFisher). 1,000× sub-stock of sorafenib (in DMSO) dissolved in BHI broth was serially diluted in 0.1% DMSO containing BHI media and inoculated with an equal volume of *S. pneumoniae,* TIGR4, D39, serotypes 3, and 19F cultures to an initial OD_620_ of 0.05–0.06. One hundred microliters of each was added in triplicate into a flat-bottom 96-well plate (Tarsons) and sealed using an optically transparent sheet (Tarsons). The plate was incubated at 37°C and OD_620_ was measured at 20 min intervals for 18 h. Bacteria incubated in plain BHI and 0.1% DMSO containing BHI were used as untreated and solvent controls, respectively. For CFU assays, the bacterial cultures were grown in the presence of sorafenib, and the cultures were serially diluted and plated on blood agar once the untreated culture reached mid-log phase (OD = 0.5).

### MIC determination in clinical strains

The minimum inhibitory concentration (MIC) of sorafenib and the comparators (penicillin, erythromycin, and DMSO) were determined by the broth microdilution method as recommended by the Clinical Laboratory Standard Institute (CLSI) (M07, 2019). Quality control strain *S. pneumoniae* ATCC 49619 was included in each run. Six clinical strains with varying penicillin and erythromycin MIC values, as listed in Table 1 were used for MIC testing. Briefly, the bacterial inoculum (10^5^ CFU/mL) was prepared in cation-adjusted Mueller-Hinton Broth (CAMHB) with 2.5% lysed horse blood (LHB). The 100 mM sorafenib stock (46.46 mg/mL) and the comparator antimicrobials were diluted in CAMHB with LHB to get 128 μg/mL concentration and then serially diluted in the microtiter plate up to 0.06 μg/mL. Equal volume of the inoculum (50 μL) was added to each well containing the serially diluted drugs in a final volume of 100 μL. The bacterial concentration of the inoculum that was added to the titer plate was calculated by performing serial dilution from the growth control. The plates were incubated at 37°C for 20–24 h. The wells were tested the next day for the inhibition of growth and the viability (bactericidal effect). The MIC value of sorafenib/antibiotic is taken as the lowest concentration that shows the visible inhibition of the growth. The susceptibilities of antibiotics were interpreted following the CLSI criteria (M100, 2019). The viability was measured in CFU/mL by subculturing from four concentrations (64, 32, 16, and 8 μg/mL); one dilution above and two dilutions below the MIC, and from the MIC well onto sorafenib/antibiotic-free media the next day after incubation. To determine the CFU/mL, the serial dilutions were made from each concentration and inoculated onto blood agar plates for colony count.

### Phospho-threonine western blotting

*S. pneumoniae* D39 and T4 strains were grown at 37°C in BHI media containing DMSO and sorafenib at the required concentrations and incubated. Post 1 h of treatment, the pellets were lysed on ice for 2 h using RIPA lysis buffer supplemented with protease inhibitor cocktail and PhosSTOP (Roche). The lysate protein was quantified by the BCA Protein Assay Kit (Pierce). Twenty-five micrograms of the lysate proteins were resolved on 7.5% SDS-PAGE for StkP, 10% for MapZ and RpoA. Rabbit induced anti-*S. pneumoniae* StkP antibody at 1:200,000, MapZ antibody at 1:70,000, and RpoA antibody at 1:40,000 dilution (gifted by Dr. Pavel Branny, Czech Academy of Sciences, Czech Republic) in 1% BSA in 1× TBS with 0.1% Tween-20 were used. Mouse anti-phospho-threonine monoclonal antibody (Cell Signaling Technology) at 1:2,000 dilution was used. Goat induced anti-rabbit and anti-mouse antibodies conjugated to HRP (Bio-Rad) at 1:5,000 dilution were used as the secondary antibodies, respectively.

### Cloning and purification of StkP kinase domain

pET22b(+) vector was double digested using Hind-III and Nde-I (GeneI) restriction enzymes at 37°C for 4 h, followed by heat inactivation at 65°C for 20 min. The digested product was resolved in 1% agarose gel and isolated by gel extraction using Macherey-Nagel kit. StkP kinase domain comprising 1–277 amino acids (NCBI: NC_003028.3) was amplified using the primers (Forward: GATATACATATGatccaaatcggcaagatt; Reverse: GGCCGCAAGCTTacgattgtaggacaagctacta) at an annealing temperature of 55°C. The gel purified PCR products were double digested using Hind-III and Nde-I, followed by clean up. Ligation was performed in the insert to vector molar ratio 15:1, using T4 DNA ligase at 16°C for 3 h. The ligated product was transformed into *E. coli* Mach1 cells. Colonies were screened by colony PCR using the above set of primers. Plasmids were isolated from positive colonies, and the clone was confirmed by double digestion as well as Sanger sequencing. The product consists of the C-terminal 6×-His tag region followed by 1–277 residues of pneumococcal StkP-kinase domain (KDC2 plasmid).

For protein expression, the plasmid construct was transformed into *E. coli* BL21(DE3) cells and plated onto an LB agar plate with 100 μg/mL of ampicillin. Primary cultures were grown in 15 mL LB medium supplemented with 100 μg/mL of ampicillin for 12 h at 30°C and 200 rpm, followed by secondary culture to OD_600_ of 0.7. The culture was then induced using 1 mM IPTG at 18°C for 16 h. The bacterial pellet was lysed using 50 mM Tris-HCl (pH 7.4), 500 mM NaCl, 10 mM imidazole, 1 mM phenylmethylsulfonyl fluoride (PMSF), 1 mM benzamidine hydrochloride, and 3 mM β-mercaptoethanol, followed by sonication for 20 min at 40% amplitude with 3 s on and 6 s off cycles. The soluble fraction was collected by a high-speed spin at 20,000 × *g* for 45 min at 4°C. The supernatant was filtered using a 0.45 μm syringe filter. The filtered supernatant was used for the affinity chromatography.

For affinity purification of the protein, an EconoFit Nuvia IMAC Ni-NTA column of 5 mL volume (Bio-Rad) connected to an NGC Quest 10 protein purification system (Bio-Rad) was used. The column was pre-equilibrated with 8× column volume of the lysis buffer, followed by sample injection. The column was then washed with 8× column volume of a wash buffer (20 mM Tris-HCl [pH 7.4], 300 mM NaCl, 25 mM imidazole, 1 mM PMSF, 1 mM benzamidine hydrochloride, and 3 mM β-mercaptoethanol), followed by elution with 10× column volume of an elution buffer containing 500 mM imidazole. The peak fractions were loaded on a 12% SDS-PAGE, and the protein fractions were combined and concentrated to 5 mL using a Vivaspin centrifugal concentrator with 10,000 MWCO (Sartorius). The supernatant was used for the size-exclusion chromatography step using a HiPrep16/60 Sephacryl HR S-200 column (Cytiva), pre-equilibrated with 1× column volume of a size-exclusion buffer containing 20 mM Tris-HCl (pH 7.4), 300 mM NaCl, 1 mM EDTA, and 1 mM DTT, and connected to an NGC Quest 10 protein purification system. The peak fractions were analyzed on a 12% SDS-PAGE, and the pure fractions were pooled together and concentrated. The concentrated protein sample was dialyzed into a buffer containing 25 mM HEPES, 300 mM NaCl, and 10 mM MgCl_2_ (pH 7.4) and was aliquoted, flash-frozen in liquid nitrogen, and stored in −80°C. The purity of the purified protein was checked by anti-His western blotting (Sigma; 1:2,000 dilution).

### *In vitro* autophosphorylation and kinase glo luminescence assay

*In vitro* phosphorylation of the purified StkP-KD was performed as described previously (54) with minor modifications. Briefly, 1 µM of purified StkP-KD was incubated in buffer containing 50 mM HEPES (pH 7.4), 1 mM DTT, 0.01% Tween-20, and 20 mM MgCl_2_ containing ATP and incubated at 37°C for 30 min. The reaction was stopped with 4× Laemmli sample buffer (Bio-Rad) containing 50 mM DTT (HiMedia), followed by boiling at 70°C for 10 min. The samples were resolved on a 10% SDS-PAGE, and western blotting with anti-phospho-threonine monoclonal antibody (Cell Signaling Technology) was performed as described previously. A gradient of ATP concentrations (10–500 µM) was used to study dose dependency. The kinase reaction was performed in the presence of ATP (250 µM), sorafenib (250, 500, and 1,000 µM), or equivalent DMSO (5%) as the solvent control.

The Kinase-Glo Luminescent Assays (Promega) were performed in 50 µL kinase assay buffer, as described above. Sorafenib stock was diluted in DMSO into 100× of experimental concentrations, effectively making the final DMSO to be 1% in all samples. The kinase reaction was performed in the presence of 10 µM ATP. The reaction mix was equilibrated to room temperature post 30 min of kinase reaction at 37°C, and mixed with an equal volume of Kinase GloMax reagent (Promega) in a white opaque 96-well plate (Tarsons), and the luminescence at 562 nm was acquired post 15 min of incubation at room temperature using a Varioskan LUX Spectrophotometer (Thermo Scientific) with an integration time of 500 ms.

### Isothermal titration calorimetry (ITC)

The purified StkP-KD protein was extensively dialyzed into a buffer containing 25 mM HEPES, 300 mM NaCl, 10 mM MgCl_2_ and 1% DMSO (pH 7.4). For titrations, sorafenib was diluted in the dialysis buffer to a final DMSO concentration of 1% in all the samples. ITC experiments were performed using a Malvern MicroCal PEAQ ITC machine at the Department of Biotechnology, Indian Institute of Technology (IIT), Madras. Protein and drug samples were degassed by vacuum aspiration for 5–10 min at room temperature prior to loading the samples in the ITC cell and syringe. All titrations were carried out at 25°C with a stirring speed of 500 rpm and a 360 s duration between each 2 μL injection. Buffer-to-buffer titrations and ligand-to-buffer titrations were used as the controls. Ligand to protein titration was done at 80× molar ratio (400 µM SFN to 5 µM StkP-KD). The heats of dilution were negligible in all control experiments and were subtracted from their respective titrations prior to data analysis. The thermodynamic parameters *N* (stoichiometry), *K*_A_ (association constant), and Δ*H* (enthalpy change) were obtained by fitting of experimental data using the sequential binding sites model using the instrument software. The free energy of binding (Δ*G*) and entropy change (Δ*S*) were obtained using the equations $\Delta G=-RTlnk_{A}$ and ΔG=ΔH-TΔS, respectively. The binding affinities of sorafenib to StkP-KD for all the binding sites are given as the dissociation constants (*K*_*D*_ = 1/*K*_*A*_). Titration data were analyzed independently, and the thermodynamic values obtained were indicated.

### A549 infection assay

*S. pneumoniae* TIGR4-GFP strain was grown on BHI agar plates containing chloramphenicol (CmR). The cultures in BHI-CmR broth were treated with 10 µM Sorafenib and DMSO and grown at 37°C to an OD_600_ of 0.4. Based on the CFU count of 3.55 × 10^7^ CFU/mL, the volume of bacterial suspension containing 2 × 10^7^ cells/well for multiplicity of infection (MOI) of 100:1 was washed and resuspended in 50 µL of PBS. A549 cells were seeded in a 12-well plate at a density of 2 × 10^5^ cells/well in 750 µL Dulbecco’s modified Eagle medium (DMEM) (HiMedia) supplemented with 10% fetal bovine serum (FBS) (Invitrogen) and incubated overnight at 37°C and 5% CO_2_. Untreated, DMSO-treated, and sorafenib-treated bacteria were added to A549 cells in wells containing 750 µL DMEM supplemented with 5% heat-inactivated FBS without antibiotics. The plate was centrifuged at 1,500 rpm for 5 min to enhance bacterial contact and incubated at 37°C, 5% CO_2_ for 3 h. After incubation, the medium was aspirated gently and A549 cells were harvested by trypsinization. The cells were washed and fixed at room temperature for 10 min with 4% paraformaldehyde. The cells were analyzed using the BD FACS Aria III cytometer (BD Biosciences) in the FITC channel to detect GFP signals.

### Live-dead staining of bacteria

Live-dead staining was performed using the BacLight Bacterial Viability Kit (ThermoFisher), following the manufacturer’s protocol. Bacteria were grown to mid-log phase in the presence of 10 μM Sorafenib or equivalent DMSO. Approximately 5.8 × 10^7^ CFU/mL of bacterial cells were washed and resuspended in 500 μL PBS. Each sample was incubated with 1.5 μL of the staining dye mix containing equal volumes of SYTO9 and Propidium Iodide for 15 min under dark conditions. The cells were washed twice in PBS again, followed by fixation with 4% paraformaldehyde (HiMedia) at room temperature for 10 min. Bacteria treated with 70% ethanol (Molecular Biology Grade, Supelco) for 15 min at room temperature were used as the positive control of death. The fixed cells were mounted (ProLong Glass Antifade Mountant, Invitrogen) on glass slides, using No. 1 English Coverslips (Bluestar) and visualized using a Nikon Eclipse Ti2 Inverted Confocal Imaging System.

### Scanning electron microscopy

The bacteria were grown in BHI broth at 37°C and 5% CO_2_ to an OD_600_ of 0.7. The bacteria were then pelleted and washed thrice with PBS, following fixation with 4% paraformaldehyde at room temperature for 2 h. After 3 washes in distilled water, sequential dehydration was followed using 30% ethanol for 10 min, 50% ethanol for 10 min, 70% ethanol for 10 min, and 100% ethanol for 15 min. Subsequently, the pellet was lyophilized by dehydration using a speed vacuum at 30-s intervals for 3 min and then air dried in a biosafety cabinet for 1–2 h. The adhesive carbon tape (PELCO Tabs, 12 mm OD, 16084-1, Ted Pella) was placed on the sample holders, and the powdered sample was dusted on the tape using a paintbrush. The gold sputtering was done using a QT Quorum Sputter Coater with a thickness of about 10 nm. Gold-coated specimens were imaged using FEI Nova Nanosem 450 at the Indian Institute of Science Education and Research, Thiruvananthapuram, under a high vacuum at 15.0 kV, with a 5.6 mm working distance and a 40 μm objective lens aperture. Images were collected using the secondary electron detector; the acquisition time per image was 10–20 μs, and each image was 1,536 × 1,103 pixels. SEM images were recorded at magnifications ranging from 10,000× to 100,000×.

### Complement deposition and serum killing assay

Briefly, *S. pneumoniae* TIGR4 strain was grown to mid-log phase in BHI media. Untreated and 10 μM DMSO treated bacteria grew to OD_600_ of 0.5, while the 10 μM sorafenib treated bacteria maintained a static OD_600_ of 0.1. Upon OD normalization of the cultures to 0.5 at OD_600_, ~5.8 × 10^7^ CFU/mL of bacterial cells were washed with PBS buffer and incubated with 10% healthy donor serum (diluted in PBS) at 37 °C for 30 min. Bacteria incubated with heat-inactivated human serum served as the negative control. As a positive control, the encapsulated TIGR4 strain was permeabilized using 0.05% Triton X-100 (HiMedia) and subsequently opsonized with an anti-*Streptococcus pneumoniae* antibody (ab20429; Abcam) for 30 min at 37°C. The bacterium-bound C3 protein was stained with goat-induced anti-C3 antibody (Sigma-Aldrich, 204869) at 1:100 dilution, followed by an incubation with Alexa Fluor 488 conjugated donkey-induced anti-goat secondary antibody (Invitrogen, A-11055) at 1:400 dilution. The bacteria were blocked with 1% BSA (HiMedia) in PBS before antibody incubation, and all steps had one PBS wash in between. Finally, the samples were fixed with 2% paraformaldehyde (HiMedia) and resuspended in PBS. The samples were stored at 4°C till analyzing them by flow cytometry (CytoFlex S, Beckman Coulter). The data were analyzed using Kaluza version 2.2.1.

For serum killing, the bacterial cells incubated with serum were washed and resuspended in 100 µL PBS, followed by serial dilution and CFU enumeration on blood agar plates.

### Serial passaging assay for drug resistance

Pneumococci were continuously passaged on plates containing 1 μM (sub-MIC) and 2.5 μM (MIC) concentrations of sorafenib to study resistance development. For sub-MIC passaging experiments, 50 μL of OD normalized pneumococci after pre-treatment with 1 μM sorafenib in BHI broth (passage 0) were plated on BHI plates containing 1 μM and 2.5 μM of sorafenib (passage 1). After overnight incubation at 37°C and 5% CO_2_, the plates were assessed for growth. Colonies grown on sorafenib-1 μM plates were harvested into 2 mL of BHI broth to create a primary suspension. The primary suspension was OD-normalized to 0.5, and 50 µL was subsequently plated onto 1 μM and 2.5 μM sorafenib plates (passage 2), and the remaining suspension was frozen. For initial passages under 1 μM sorafenib showing minimal growth (and thus undetectable OD), one-fifth of the primary suspension was directly plated. To evaluate survival rates, OD-normalized pneumococci treated with 1 µM sorafenib were serially diluted and plated on blood agar. Colonies obtained from 50 µL of this dilution were counted and defined as the input CFU. For output CFU determination, 100 µL of the primary suspensions were serially diluted and plated on blood agar. The procedure was carried out for 31 passages.

For MIC passaging experiments, one-fourth of the primary suspension obtained from SFN-2.5 μM plates was directly plated without OD normalization due to the negligible growth on the plates. Plating was continued for four passages until no colony growth was observed, indicating minimal resistance.

### Mouse experiments

C57BL/6J mice were generated and maintained at the Animal Research Facility, BRIC-RGCB using Individually Ventilated Caging (IVC) system with 14 h light and 10 h darkness at 25°C with *ad libitum* access to food and water. Mice were used at the age of 6–9 weeks. The mice were randomly assigned to the experimental groups and included a minimum of five mice/group.

Six- to nine-week-old wild-type male C57BL/6 were anesthetized by inhalation of 5% isoflurane for 5 min and oropharyngeally administered with 1 × 10^6^ CFU of T4 strain at mid-log phase, resuspended in 50 μL of 1× endotoxin-free PBS (Invitrogen). Mice were randomly allotted to treatment and control groups (>10 mice/group). Briefly, 10 mg/kg of sorafenib in 55% PEG-400 (Sigma-Aldrich) and 20% DMSO (HiMedia) was administered by tail vein injection post 1 h of infection and intraperitoneally every 24 h till the ethical end point was attained. Mice treated with PEG-400 and DMSO alone served as the placebo control. Animals were clinically scored every 24 h for the symptoms and were sacrificed upon reaching the ethical end points. Euthanasia was done by inhalation of 10% CO_2_ and cardiac puncture was performed post euthanasia to collect the blood sample. Post sacrifice, cardiac perfusion was performed using endotoxin-free PBS supplemented with 5 mM EDTA (HiMedia) to drain out the blood. Lungs were collected in PBS, homogenized, and strained using 70 μm cell strainers (HiMedia). One hundred microliters of the lung tissue homogenate was serially diluted and plated on blood agar. Colonies were counted post 16 h of incubation at 37°C and 5% CO_2_.

### Statistical analysis

Data were statistically analyzed using GraphPad Prism v10.4.2. Data represent mean ± SEM. Experiments were performed with three biological replicates. The exact number of biological replicates (*N*) is mentioned in the respective figure legends. Normality was calculated using the Shapiro-Wilk test and used for the statistical tests. Pairwise comparison of normalized data were analyzed using paired/unpaired *t*-tests as mentioned in the figure legends. Two-way ANOVA was used to compare across multiple groups with Tukey’s *post hoc* test. Differences were considered significant at ∗, *P* ≤ 0.05; ∗∗, *P* ≤ 0.01; ∗∗∗, *P* ≤ 0.001; ∗∗∗∗, *P* ≤ 0.0001; and ns denotes not significant.

## SUPPLEMENTAL MATERIAL

Supplemental material mbio.00618-26-s0001.pdf Supplemental figures and methods; captions for supplemental tables and videos.

Table S1 mbio.00618-26-s0002.xlsx Kinase inhibitors used for virtual screening against pneumococcal StkP-KD.

Table S2 mbio.00618-26-s0003.xlsx Docking scores of compounds with StkP-KD from virtual screening and their PubChem IDs. Docking scores for different conformers of the ligands are shown, and those with the highest docking scores were selected.

Video S1 mbio.00618-26-s0004.mp4 Interaction of sorafenib with the active site residues of pneumococcal StkP-KD.

Video S2 mbio.00618-26-s0005.mp4 Zoomed view of the catalytic cleft in Video S1.

Video S3 mbio.00618-26-s0006.mp4 Drifting of sorafenib away from the catalytic cleft upon mutating the catalytic residues Arg-135 and Asp-136 to alanine.

Video S4 mbio.00618-26-s0007.mp4 Interaction of sorafenib with the catalytic residue Asp-136 in the presence of DivIVA peptide.

Video S5 mbio.00618-26-s0008.mp4 Zoomed view of the catalytic cleft of StkP-KD in Video S4.

ASM does not own the copyrights to Supplemental Material that may be linked to, or accessed through, an article. The authors have granted ASM a non-exclusive, world-wide license to publish the Supplemental Material files. Please contact the corresponding author directly for reuse.
