# Supplementary material for: Sorafenib, a clinical kinase inhibitor, attenuates Streptococcus pneumoniae pathogenesis and reduces disease progression in vivo
Source: mBio. 2026 Jun 16;17(7):e00618-26. doi: 10.1128/mbio.00618-26 (PMC13343887; doi:10.1128/mbio.00618-26)
Supplement: Supplemental material — Supplemental figures and methods; captions for supplemental tables and videos. [file mbio.00618-26-s0001.pdf]

Supplementary methods and data

Sorafenib, a clinical kinase inhibitor attenuates *Streptococcus pneumoniae* pathogenesis and reduces disease progression *in vivo*

Joel Abraham[1], Aswathy C. Sagilkumar [1,2], Himani Dhyani[1,2], Charmi M. Panchal[1], Shaheena Aziz[1], Priyadatha M K[1], Aan Ruth[3], Keerthana Bhaskaran[3], Sivakumar Krishnankutty Chandrika[4], Shaima S[5], Rosemol Varghese[6], Ayyanraj Neeravi[6], Balaji Veeraraghavan[6], Nagarjun Narayanaswamy[5], Sandhya Ganesan[3], Karthik Subramanian[1,2,*]

[1] Host-Pathogen Laboratory, Pathogen Biology Division, BRIC-Rajiv Gandhi Centre for Biotechnology (BRIC-RGCB), Thiruvananthapuram, 695014, India.

[2] Regional Centre for Biotechnology, Faridabad, 121001, India

[3] School of Biology, Indian Institute of Science Education and Research, Thiruvananthapuram, 695551, India

[4] Bioinformatics Facility, BRIC-RGCB, Thiruvananthapuram, 695014, India

[5] Transdisciplinary Biology Program, BRIC-Rajiv Gandhi Centre for Biotechnology, BRIC-RGCB, Thiruvananthapuram, 695014, India

[6] Department of Clinical Microbiology, Christian Medical College, Vellore, Tamil Nadu, 632004, India

[*] Correspondence to lead contact, Dr. Karthik Subramanian, Scientist E1, Host-Pathogen Laboratory, BRIC-Rajiv Gandhi Centre for Biotechnology (BRIC-RGCB), Thiruvananthapuram, 695014, India.

Email: karthik@rgcb.res.in

Tel: +91-471-2764052

Supplementary figures

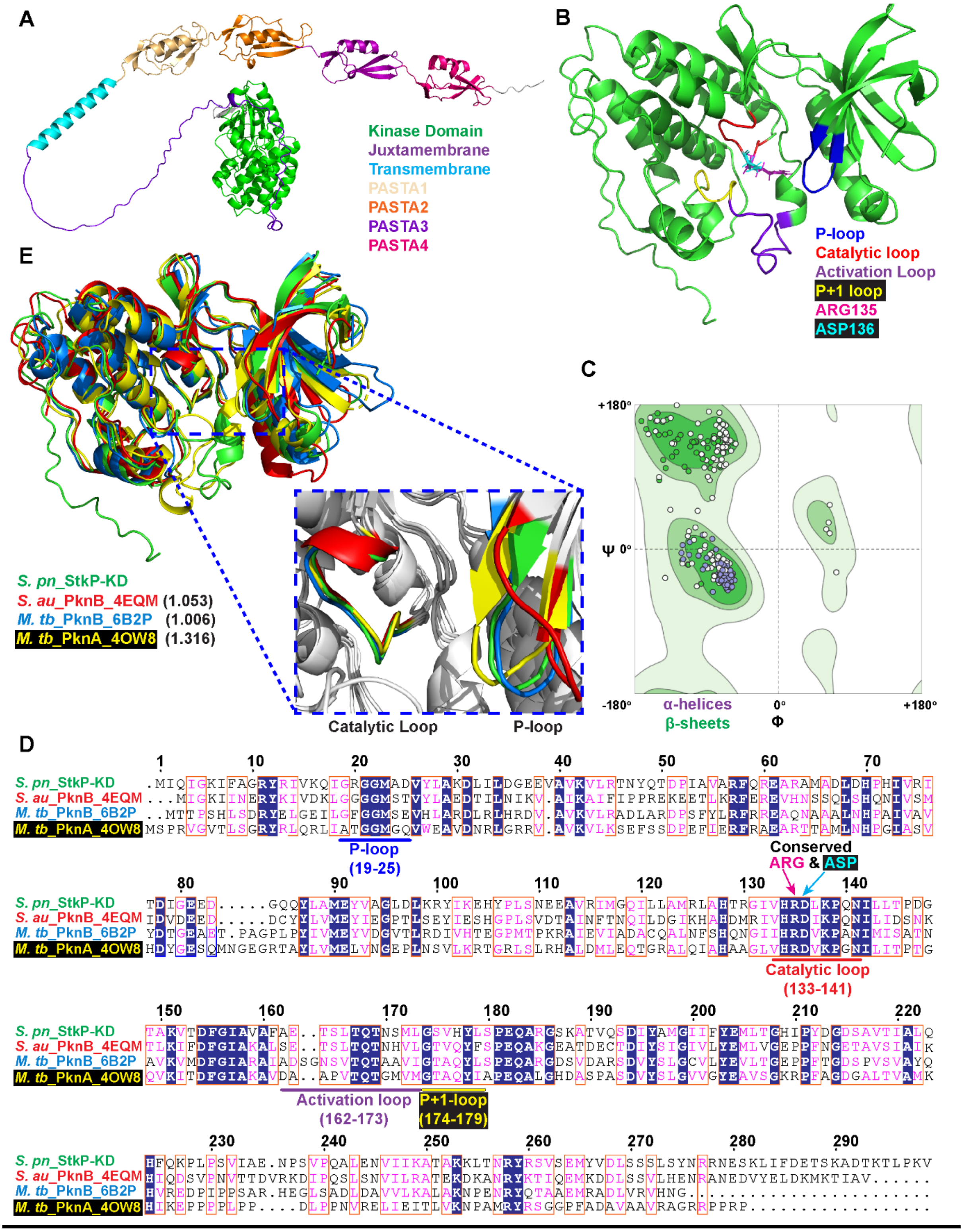

FIGURE S1: Structure modelling and validation of pneumococcal StkP-kinase domain (Related to Figure 1). **(A).** Structure of full-length StkP predicted by AlphaFold showing the functional domains. **(B).** Modelled structure of the kinase domain of StkP (StkP-KD) showing catalytically active regions. **(C).** Ramachandran plot of StkP-KD generated using the structure validation tool of SWISS-MODEL, a homology modelling server, **(D).** Sequence and **(E)** structural alignment diagram showing the conservation of the ATP-binding motif, P-loop and catalytically active, C-loop of StkP-KD of *S. pneumoniae* with homologues in *S. aureus (*PknB*)* and *M. tuberculosis (*PknA, PknB*)*, respectively. The PDB ID and RMSD of alignment are highlighted. Zoomed in region shows the structural similarity of catalytic and P-loop regions showing a good structural overlap.

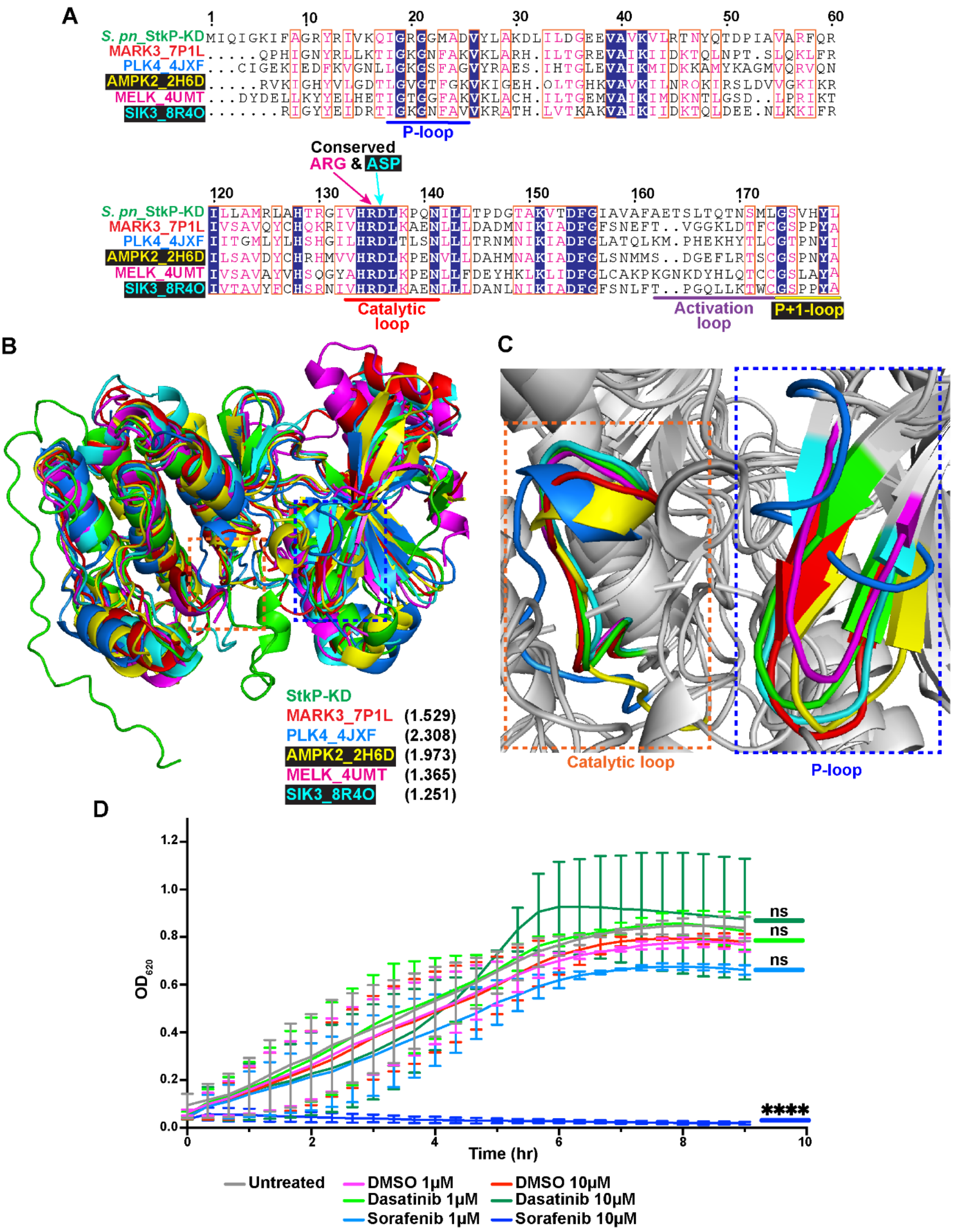
A
S. pn_StkP-KD MIQIGKIFAGRYRIVKQIGRGGMADVYLAKDLILDGEEVAVKVLRTNYQTDPIAVARFQR
MARK3_7P1L .....QPHIGNYRLLKTIGKGNFAKVKLARH.ILTGREVAIKIIDKTQLNPT.SLQKLFR
PLK4_4JXF ...CIGEKIEDFKVGNLLGKGSFAGVYRAES.IHTGLEVAIKMIDKKAMYKAGMVQRVQN
AMPK2_2H6D .....RVKIGHYVLGDTLGVGTFGKVKIGEH.QLTGHKVAVKILNRQKIRSLDVVGKIKR
MELK_4UMT ...DYDELLKYYELHETIGTGGFAKVKLACH.ILTGEMVAIKIMDKNTLGSD..LPRIKT
SIK3_8R4O .......RIGYYEIDRTIGKGNFAVVKRATH.LVTKAKVAIKIIDKTQLDEE.NLKKIFR
P-loop
Conserved
ARG & ASP
S. pn_StkP-KD ILLAMRLAHTRGIVHRDLKPQNILLTPDGTAKVTDFGIAVAFAETSLTQTNSMLGSVHYL
MARK3_7P1L IVSAVQYCHQKRIVHRDLKAENLLLDADMNIKIADFGFSNEFT..VGGKLDTFCGSPPYA
PLK4_4JXF IITGMLYLHSHGILHRDLTLSNLLLTRNMNIKIADFGLATQLKM.PHEKHYTLCGTPNYI
AMPK2_2H6D ILSAVDYCHRHMVVHRDLKPENVLLDAHMNAKIADFGLSNMMS..DGEFLRTSCGSPNYA
MELK_4UMT IVSAVAYVHSQGYAHRDLKPENLLFDEYHKLKLIDFGLCAKPKGNKDYHLQTCCGSLAYA
SIK3_8R4O IVTAVYFCHSRNIVHRDLKAENLLLDANLNIKIADFGFSNLFT..PGQLLKTWCGSPPYA
Catalytic loop
Activation loop
P+1-loop
B
StkP-KD
MARK3_7P1L (1.529)
PLK4_4JXF (2.308)
AMPK2_2H6D (1.973)
MELK_4UMT (1.365)
SIK3_8R4O (1.251)
C
Catalytic loop
P-loop
D
OD620
Time (hr)
ns
ns
ns
****
Untreated
DMSO 1μM
DMSO 10μM
Dasatinib 1μM
Dasatinib 10μM
Sorafenib 1μM
Sorafenib 10μM

FIGURE S2: P-loop and C-loop conservation in bacterial and human kinases (Related to Figure 1). **(A).** Sequence and (**B).** structural alignment diagrams showing the conservation of P-loop and Catalytic loop (C-loop) of pneumococcal eukaryotic type Ser/Thr kinase domain, StkP-KD and human kinases with available PDB structure, that showed the highest similarity. Microtubule affinity-regulating kinase 3 (MARK3; 7P1L), polo-like kinase 4 (PLK4; 4JXF), AMP-activated protein kinase catalytic subunit alpha-2 (AMPK2; 2H6D), Maternal embryonic leucine zipper kinase (MELK; 4UMT), Salt-Inducible Kinase 3 (SIK3; 8R4O). **(C)**. The C-loop and P-loop regions are magnified to show structural overlap. **(D).** Growth kinetics of *S. pneumoniae* TIGR4 strain in the presence of the top two kinase inhibitor compounds from docking studies, sorafenib and dasatinib at 1 μM and 10 μM doses. **** indicates $p \leq 0.0001$ and ns denotes non-significance with respect to equivalent DMSO (solvent) concentrations by Mann-Whitney test. Data is representative of two independent experiments.

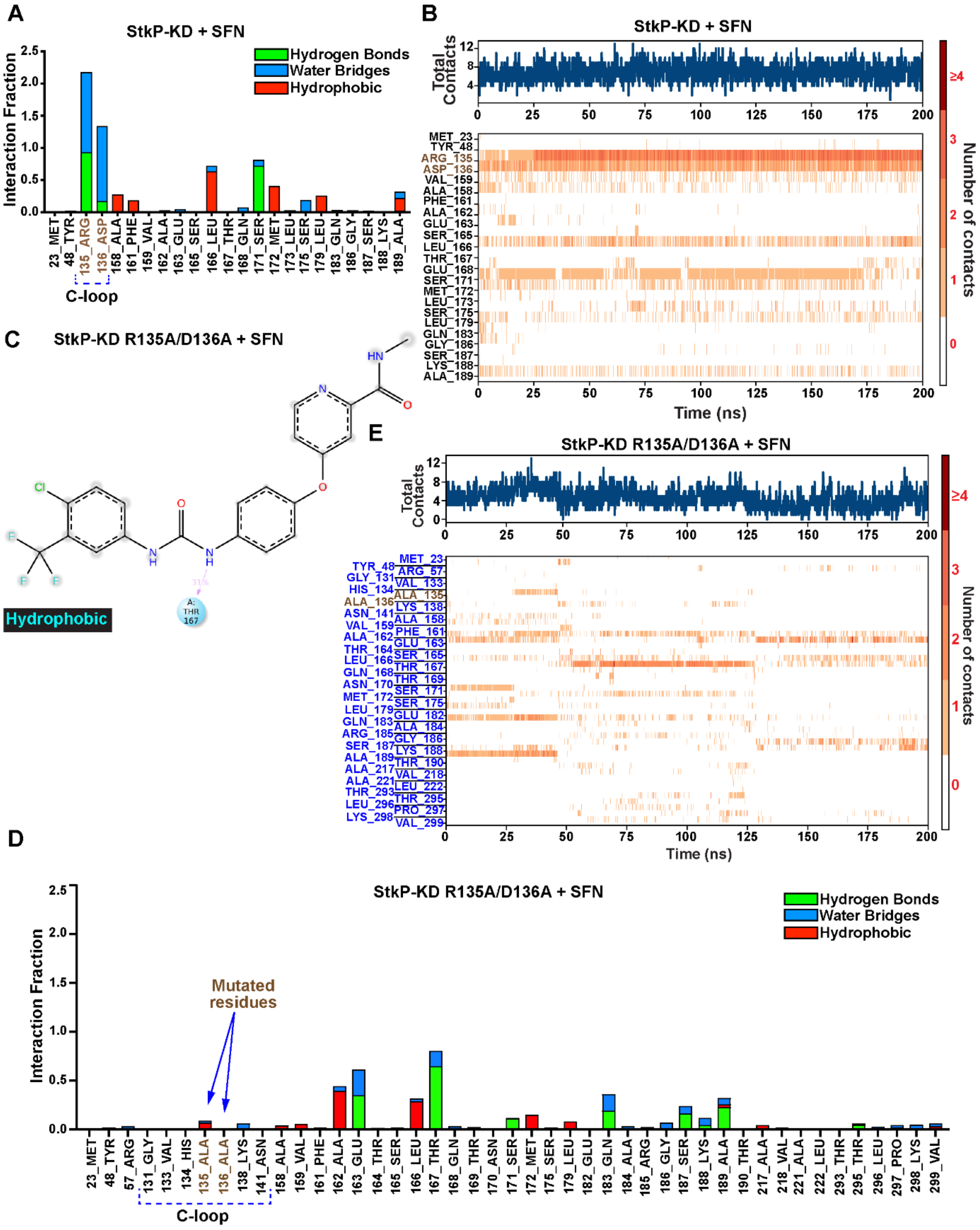
A
StkP-KD + SFN
Hydrogen Bonds
Water Bridges
Hydrophobic
Interaction Fraction
C-loop
B
StkP-KD + SFN
Total Contacts
Time (ns)
Number of contacts
≥4
C
StkP-KD R135A/D136A + SFN
Hydrophobic
E
StkP-KD R135A/D136A + SFN
Total Contacts
Time (ns)
Number of contacts
D
StkP-KD R135A/D136A + SFN
Hydrogen Bonds
Water Bridges
Hydrophobic
Interaction Fraction
Mutated residues
C-loop

FIGURE S3: Interaction of sorafenib with StkP-KD and StkP-KD R135A/D136A (Related to Figure 1). **(A).** Interaction fraction graph showing the time fraction of hydrogen bonds, water bridges and hydrophobic interactions between sorafenib (SFN) and the corresponding amino acids of StkP-KD during a molecular dynamic simulation for 200 ns. Catalytic loop is highlighted, and the active site residues are shown in brown. **(B).** Timeline graph showing the quantification of time-dependent contacts between SFN and StkP-KD during the simulation. Active site residues are represented in brown color. **(C).** Contact diagram showing interactions between SFN and StkP-KD R135A/D136A double mutant. Residues involved in bonding over 30% of the total simulation time are shown. **(D).** Interaction fraction graph showing the time fraction of hydrogen bonds, water bridges and hydrophobic interactions between SFN and StkP-KD R135A/D136A. The C-loop region is highlighted, and the mutated residues are shown in brown. **(E).** Timeline graph showing the quantification of time-dependent contacts between SFN and StkP-KD R135A/D136A during the simulation.

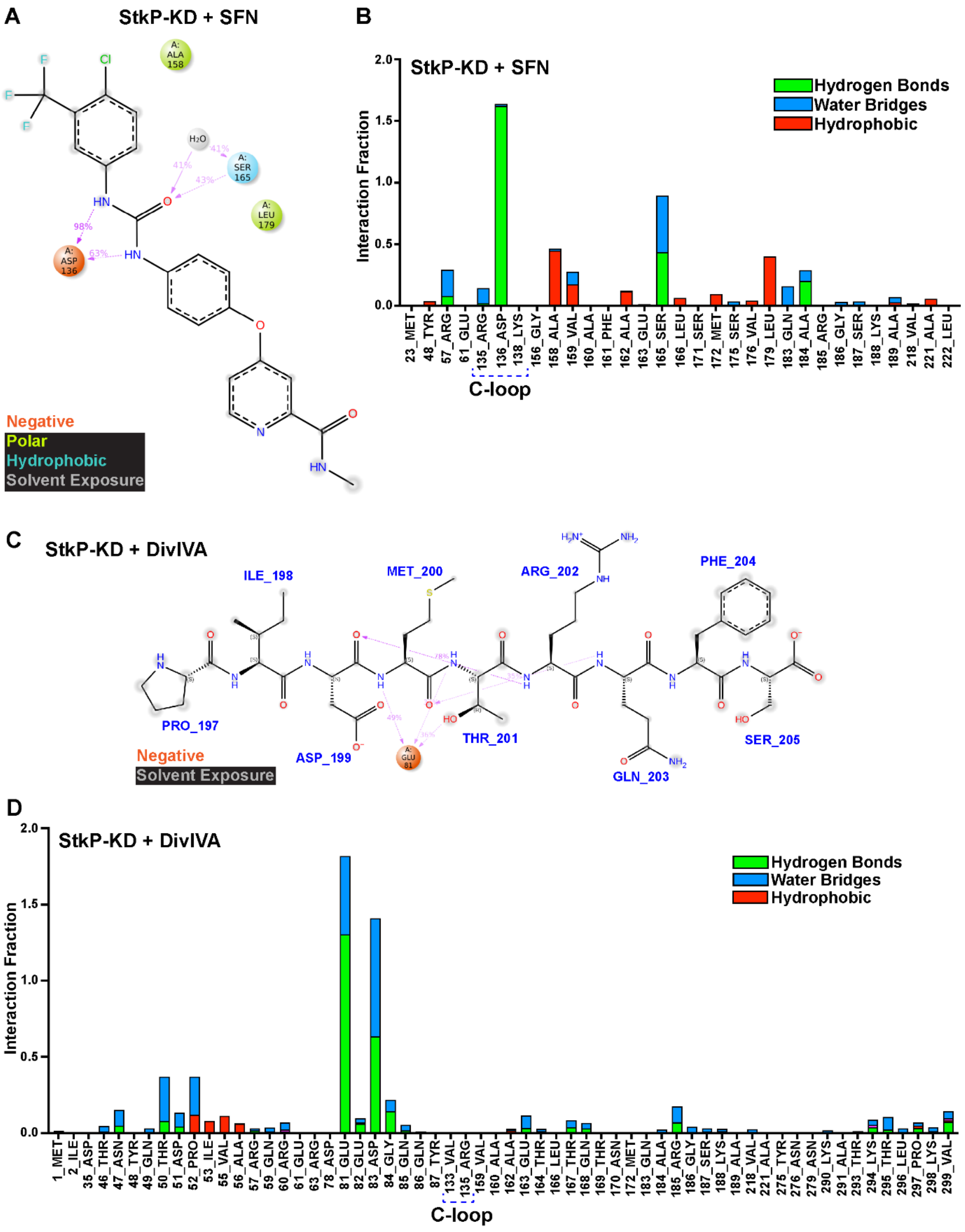
A
StkP-KD + SFN
A: ALA 158
A: SER 165
A: LEU 179
A: ASP 136
H2O
41%
41%
43%
98%
63%
Negative
Polar
Hydrophobic
Solvent Exposure
B
StkP-KD + SFN
Interaction Fraction
2.0
1.5
1.0
0.5
0.0
Hydrogen Bonds
Water Bridges
Hydrophobic
23_MET
48_TYR
57_ARG
61_GLU
135_ARG
136_ASP
138_LYS
156_GLY
158_ALA
159_VAL
160_ALA
161_PHE
162_ALA
163_GLU
165_SER
166_LEU
171_SER
172_MET
175_SER
176_VAL
179_LEU
183_GLN
184_ALA
185_ARG
186_GLY
187_SER
188_LYS
189_ALA
218_VAL
221_ALA
222_LEU
C-loop
C
StkP-KD + DivIVA
ILE_198
MET_200
ARG_202
PHE_204
PRO_197
ASP_199
THR_201
GLN_203
SER_205
A: GLU 81
Negative
Solvent Exposure
D
StkP-KD + DivIVA
Interaction Fraction
2.0
1.5
1.0
0.5
0.0
Hydrogen Bonds
Water Bridges
Hydrophobic
1_MET
2_ILE
35_ASP
46_THR
47_ASN
48_TYR
49_GLN
50_THR
51_ASP
52_PRO
53_ILE
55_VAL
56_ALA
57_ARG
59_GLN
60_ARG
61_GLU
63_ARG
78_ASP
81_GLU
82_GLU
83_ASP
84_GLY
85_GLN
86_GLN
87_TYR
133_VAL
135_ARG
159_VAL
160_ALA
162_ALA
163_GLU
164_THR
166_LEU
167_THR
168_GLN
169_THR
170_ASN
172_MET
183_GLN
184_ALA
185_ARG
186_GLY
187_SER
188_LYS
189_ALA
218_VAL
221_ALA
275_TYR
276_ASN
279_ASN
290_LYS
291_ALA
293_THR
294_LYS
295_THR
296_LEU
297_PRO
298_LYS
299_VAL
C-loop

FIGURE S4: Competitive binding of Sorafenib and DivIVA peptide with StkP-KD (Related to Figure 1). **(A).** Contact diagram of interactions between sorafenib (SFN) and StkP-KD, during a 200 ns molecular dynamic simulation in the presence of DivIVA peptide. Residues involved in bonding over 30% of the total simulation time are shown. (**B**). Interaction fraction graph showing the time fraction of hydrogen bonds, water bridges and hydrophobic interactions of SFN with StkP-KD in the presence of DivIVA peptide. Catalytic loop is highlighted, and the active site residues are shown in brown. **(C).** Contact diagram showing the interactions between DivIVA peptide and StkP-KD during the simulation in the presence of SFN. Only residues involved in bonding over 30% of the total simulation time are shown. **(D).** Interaction fraction graph showing the time fraction of interactions between DivIVA peptide and StkP-KD during the simulation in the presence of SFN. Catalytic loop is highlighted, and the active site residues are shown in brown.

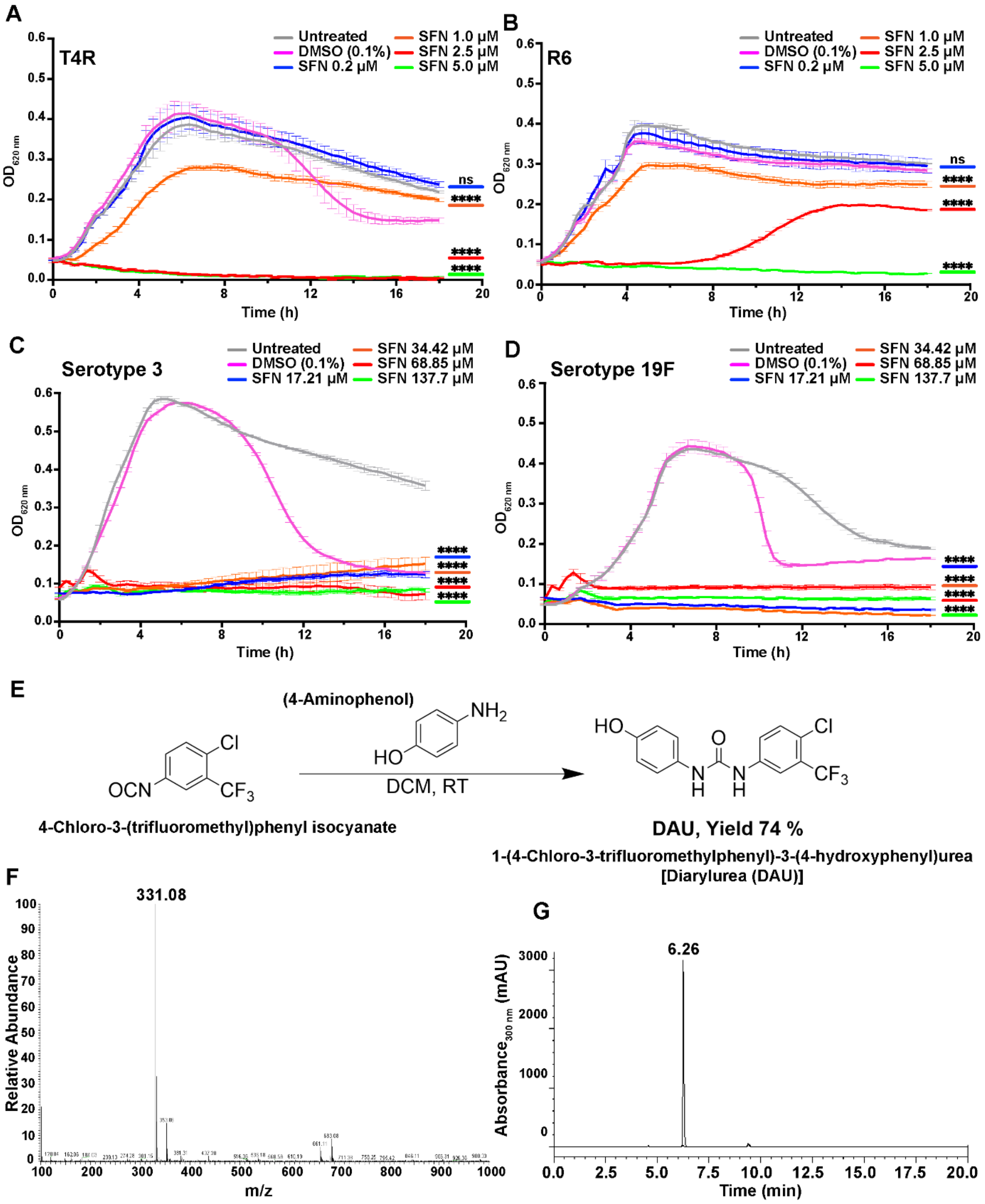
A
T4R
Untreated
DMSO (0.1%)
SFN 0.2 μM
SFN 1.0 μM
SFN 2.5 μM
SFN 5.0 μM
ns
****
OD620 nm
Time (h)
B
R6
Untreated
DMSO (0.1%)
SFN 0.2 μM
SFN 1.0 μM
SFN 2.5 μM
SFN 5.0 μM
ns
****
OD620 nm
Time (h)
C
Serotype 3
Untreated
DMSO (0.1%)
SFN 17.21 μM
SFN 34.42 μM
SFN 68.85 μM
SFN 137.7 μM
****
OD620 nm
Time (h)
D
Serotype 19F
Untreated
DMSO (0.1%)
SFN 17.21 μM
SFN 34.42 μM
SFN 68.85 μM
SFN 137.7 μM
****
OD620 nm
Time (h)
E
(4-Aminophenol)
NH2
HO
Cl
OCN
CF3
DCM, RT
HO
O
N H
N H
Cl
CF3
4-Chloro-3-(trifluoromethyl)phenyl isocyanate
DAU, Yield 74 %
1-(4-Chloro-3-trifluoromethylphenyl)-3-(4-hydroxyphenyl)urea
[Diarylurea (DAU)]
F
331.08
Relative Abundance
m/z
G
6.26
Absorbance300 nm (mAU)
Time (min)

FIGURE S5: Activity of sorafenib on *S. pneumoniae* strains and synthesis of Diarylurea (DAU) (Related to Figure 2). **(A-B).** Growth kinetics of non-encapsulated *S. pneumoniae* strains, **(A)** T4R (serotype 4) and **(B)** R6 (serotype 2), showing dose-dependent inhibition of growth with increasing concentrations of sorafenib (SFN). DMSO treated and untreated bacteria served as negative controls. **** indicates $p \leq 0.0001$ relative to untreated; ns denotes non-significance by Mann-Whitney test**. (C-D).** Growth kinetics of clinical strains, **(C)** serotype 3 and **(D)** 19F, showing the inhibition of growth by sorafenib at 17.21 to 137.7 μM. DMSO treated and untreated bacteria served as negative controls. **** indicates $p \leq 0.0001$ relative to untreated by Mann-Whitney test. Data are representative of mean ± SEM from three independent experiments. **(E).** Synthesis of 1-(4-Chloro-3-trifluoromethylphenyl)-3-(4-hydroxyphenyl) urea or Diarylurea (DAU). **(F).** MSI-ES data of the product mixture showing peak at 331.08 indicating enrichment of the DAU. **(G).** HPLC data showing a single peak indicting the purity of the final DAU product.

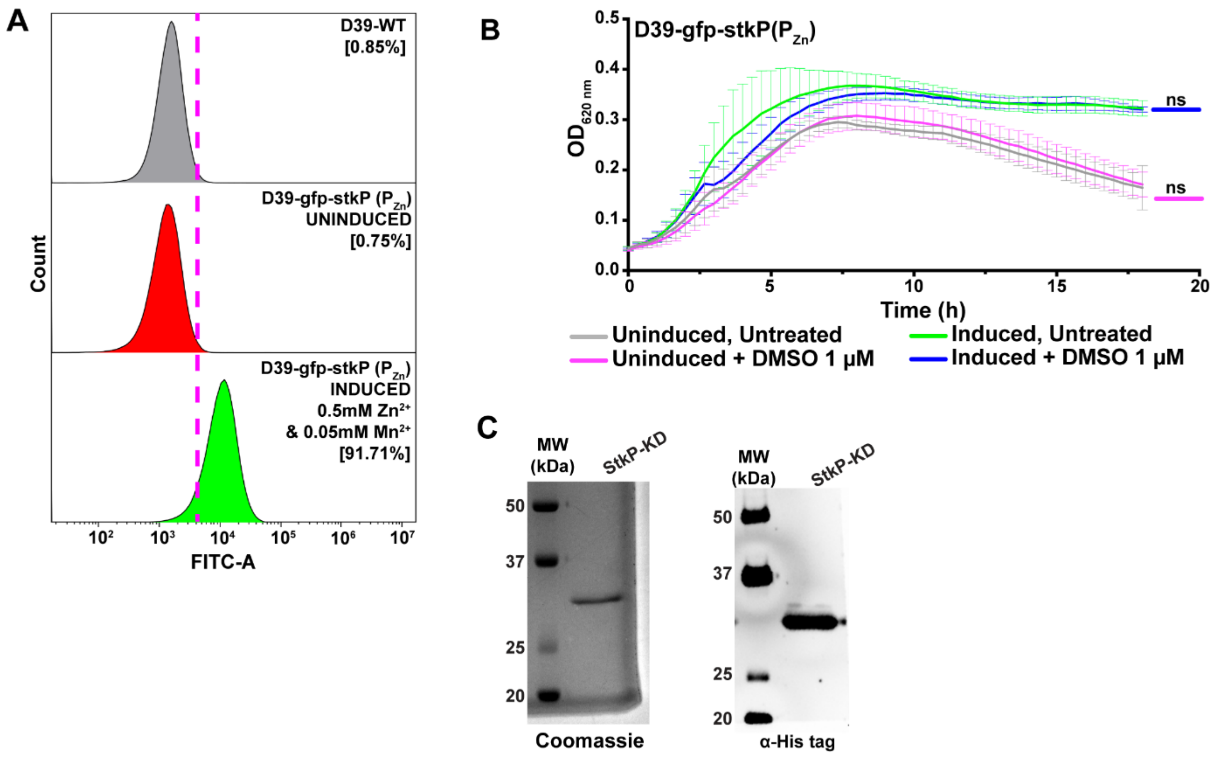

FIGURE S6: Validation of D39-gfp-stkP(P_{Zn}) strain and purified StkP-KD protein (Related to Figure 3). **(A).** Flow cytometry overlay showing the positive shift in GFP signal in D39-gfp-stkP(P_{Zn}) strain upon induction with 0.5 mM $ZnCl_2$ and 0.05 mM $MnCl_2$ compared to uninduced D39-gfp-stkP(P_{Zn}) and D39-WT strain. Percentage positivity is shown in parenthesis. **(B).** Growth kinetics of D39-gfp-stkP(P_{Zn}) in the presence 1 µM DMSO under uninduced and induced conditions. Representative of mean ± SEM from three independent experiments. ns denotes non-significance by Mann-Whitney test of DMSO relative to untreated respective to uninduced/induced conditions. **(C).** Coomassie stained gel and western blot with anti-His antibody of the purified recombinant 6x-His tagged StkP-KD protein.

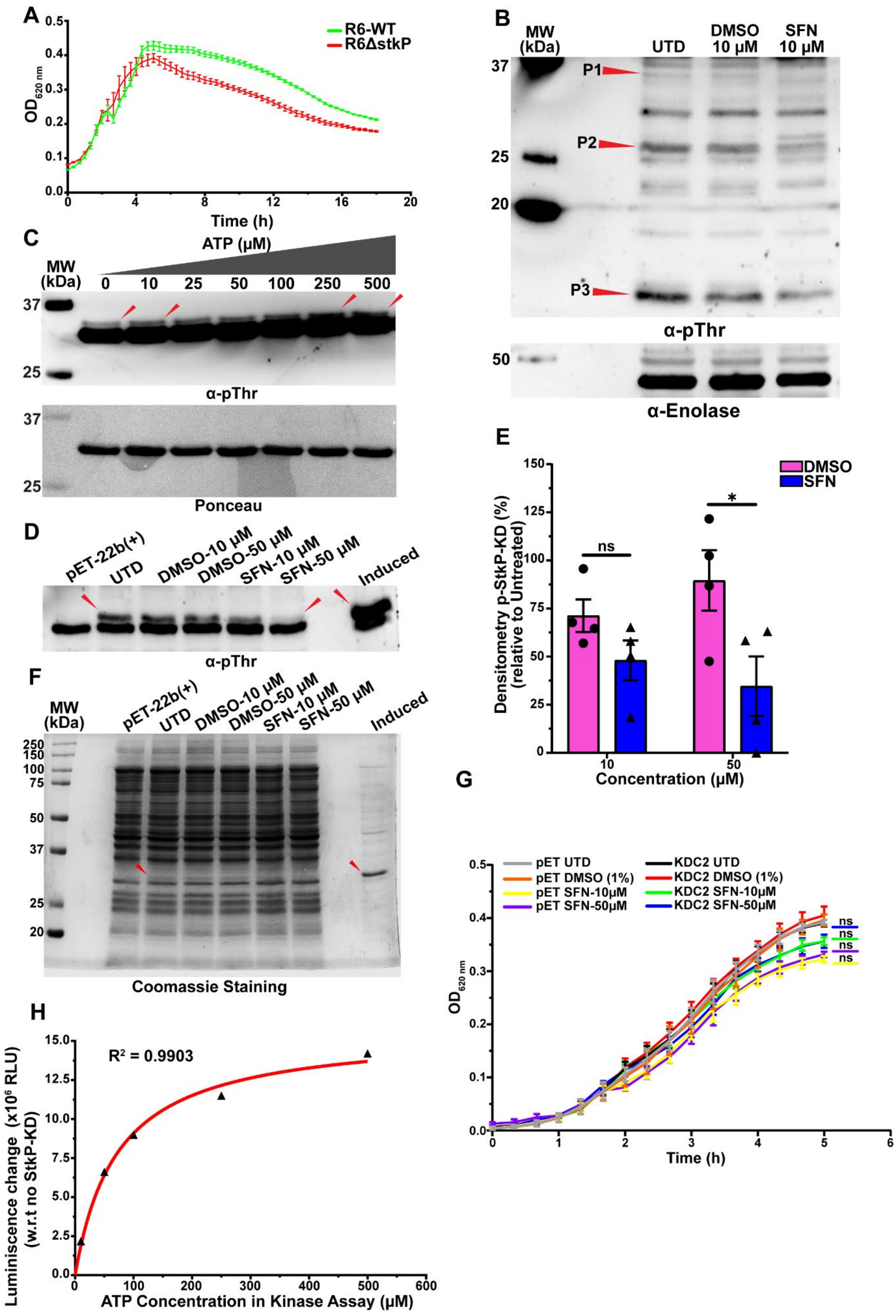

A
R6-WT
R6ΔstkP
OD 620 nm
Time (h)
B
MW (kDa)
UTD
DMSO 10 µM
SFN 10 µM
P1
P2
P3
α-pThr
α-Enolase
C
ATP (µM)
MW (kDa)
0 10 25 50 100 250 500
α-pThr
Ponceau
D
pET-22b(+)
UTD
DMSO-10 µM
DMSO-50 µM
SFN-10 µM
SFN-50 µM
Induced
α-pThr
E
DMSO
SFN
ns
*
Densitometry p-StkP-KD (%) (relative to Untreated)
Concentration (µM)
F
MW (kDa)
pET-22b(+)
UTD
DMSO-10 µM
DMSO-50 µM
SFN-10 µM
SFN-50 µM
Induced
Coomassie Staining
G
pET UTD
pET DMSO (1%)
pET SFN-10µM
pET SFN-50µM
KDC2 UTD
KDC2 DMSO (1%)
KDC2 SFN-10µM
KDC2 SFN-50µM
ns
OD 620 nm
Time (h)
H
R² = 0.9903
Luminiscence change (x10⁶ RLU) (w.r.t no StkP-KD)
ATP Concentration in Kinase Assay (µM)

FIGURE S7: Effects of sorafenib on StkP autophosphorylation activity (Related to Figure 4). **(A).** Growth kinetic assay of R6-WT and R6ΔstkP strains showing the survival of R6 strain in the absence of the StkP protein. **(B).** Western blots probed with p-Thr specific antibody and enolase (loading control) showing the downregulation of phosphorylated proteins in D39 strain upon treatment with 10 μM sorafenib (SFN). Labelled bands, which showed consistent downregulation across three independent experiments were identified by gel-based mass spectrometry. Untreated (UTD) and equivalent amount of DMSO treated bacteria were used as the controls. **(C).** Western blots showing the *in vitro* autophosphorylation of purified StkP-KD under a gradient of ATP concentrations (0-500 μM). Ponceau staining of the blots was used as loading control. **(D).** Western blot probed with p-Thr specific antibody showing the dose-dependent downregulation of phosphorylation of ectopically expressed pneumococcal StkP-KD in *E. coli* BL21(DE3) cells without induction and upon treatment with SFN. Transformed *E. coli* induced for StkP-KD expression using 0.5 mM IPTG and pET-22b(+) empty vector transformed bacteria were used as controls. Arrow indicates StkP-KD band. **(E).** Densitometry analysis of the blot in panel C normalized to the untreated protein band. * indicates $p \leq 0.05$ and ns denotes non-significance by Welch's t-test. Data are representative of four independent experiments. **(F).** Coomassie stained SDS-PAGE of the blot in the panel D, used as the loading control. Data in C-F are representative of four independent experiments. **(G).** Growth kinetic assay of empty pET-22b(+) vector and KDC2 plasmid transformed *E. coli* BL21(DE3) cells upon treatment with 10 μM and 50 μM SFN and equivalent DMSO for 5 h. ns denotes non-significance relative to untreated by Welch's t-test from 3 independent experiments. **(H).** Michaelis–Menten kinetics showing the rate of ATP consumption represented by the change in luminescence in the absence and presence of purified 1 μM StkP-KD protein under a range of ATP concentrations (0-500 μM).

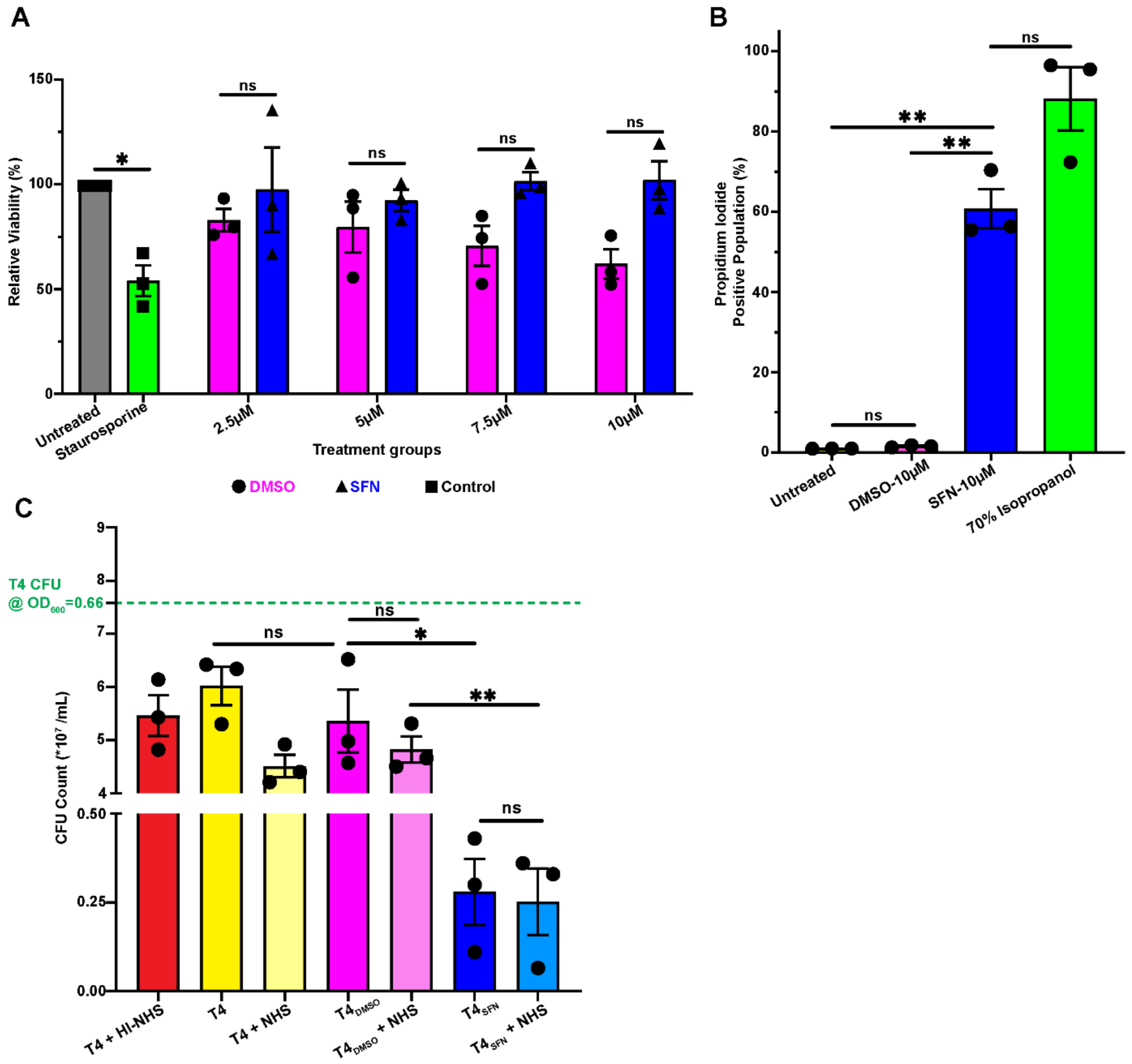

FIGURE S8: Antimicrobial effect of sorafenib with limited host cytotoxicity (Related to Figure 5). **(A).** PrestoBlue cell viability assay showing the cytotoxicity of A549 cells under 2.5 to 10 µM of sorafenib (SFN) and DMSO for 3 h. 5 µM Staurosporine-treated cells were used as the positive control for cell death. **(B).** Flow cytometry analysis showing the quantification of propidium iodide positive *S. pneumoniae* T4 upon treatment with 10 µM SFN. 70% isopropanol treated bacteria were used as the positive control of death. **(C).** CFU plating assay showing the killing of 10 µM SFN treated T4 strain upon incubation with 10% normal human serum (NHS). Bacteria treated with equimolar DMSO concentration served as the solvent control. Bacteria incubated with heat-inactivated serum was used as the negative control. * indicates $p \leq 0.05$, ** indicates $p < 0.01$ and ns denotes non-significance by paired t-test. All data represent mean ± SEM from three independent experiments.

Supplementary tables

Table S1: (Related to Figure 1 and S2). Kinase inhibitors used for virtual screening against pneumococcal StkP-KD, their CAS and PubChem IDs. Retrieved from (1).

Table S2: (Related to Figure 1 and S2). Docking scores of compounds (kJ/mol) with StkP-KD from virtual screening and their PubChem IDs. Multiple entries of the same compound indicate alternative docked confirmations. Second sheet contains the raw data file from Schrodinger.

Supplementary video legends

Video S1: Movie showing the interaction of sorafenib with the active site residues of pneumococcal StkP-KD. Sorafenib is shown as a ball and stick model with elemental colorings, Carbon in white, Oxygen in red, Nitrogen in blue, Fluorine in light green and Chlorine in dark green. StkP-KD backbone is shown as a cartoon in green color with the functionally active domains P-loop (19-25) in blue, C-loop (133-141) in red, activation loop (162-173) in purple and P+1-loop (174-179) in yellow colors respectively. The active site residues Arg-135 and Asp-136 are also represented as ball and stick model in cyan and pink colors respectively. Yellow dotted lines indicate the hydrogen bonds formed between sorafenib and catalytic residues. Movie was made using the Maestro module of Schrodinger Software Suite.

Video S2: Zoomed view of the catalytic cleft in video S1.

Video S3: Movie showing the drifting of sorafenib away from the catalytic cleft upon mutating the catalytic residues, Arg-135 and Asp-136 to alanine. Color and model representations are the same as followed in movie S1. Mutated residues Ala-135 and Ala-136 are shown in cyan and pink colors respectively.

Video S4: Interaction of sorafenib with the catalytic residue Asp-136 (cyan color) in the presence of DivIVA peptide. The peptide is shown as a stick model in orange color and Ser-165 is shown in lavender color. Rest all the representations are same as video S1.

Video S5: Movie in S4 zoomed to the catalytic cleft of StkP-KD.

Supplementary Methods

Protein preparation for docking

The predicted model of the StkP Kinase Domain was imported into the Maestro software [Schrödinger Release 2023-2: Maestro, Schrödinger, New York,]. The model was prepared using the Protein Preparation Wizard (2) [Schrödinger Release 2024-4], which involved preprocessing to add hydrogen atoms, assigning bond orders, and creating zero-order bonds to metals and disulfide bonds. Water molecules were deleted unless they were involved in critical interactions. Subsequently, hydrogen bond assignments were optimized to improve the overall structural stability. Finally, the structure underwent energy minimization to resolve any steric clashes and to refine the geometry, ensuring the model was suitable for subsequent molecular modelling studies.

Ligand preparation

The individual structures of mammalian kinase inhibitors (**Table S1**) were retrieved from the PubChem database (https://pubchem.ncbi.nlm.nih.gov/) and prepared using the LigPrep module [Schrödinger Release 2024-4: LigPrep]. The preparation followed the default settings of the tool. Briefly, the structures were processed using the OPLS4 (Optimized Potentials for Liquid Simulations) force field (3). Possible ionization states at a target pH of 7.0 ± 2.0 were generated using the Epik (Classic) program (4) [Schrödinger Release 2024-4]. The process excluded consideration of metal binding states and the original ionization state. Additionally, the structures were desalted, and tautomers were generated, with the ligand size limited to a maximum of 500 atoms. Stereoisomers were generated while retaining specified chiralities, with a limit of 32 structures per ligand. The prepared ligand structures were saved in Maestro format.

Receptor grid generation

A binding pocket identified by the SiteMap tool (5) [Schrödinger Release 2024-4], containing the active site residue Asp 136 along with other catalytic loops, was selected for grid generation for docking studies. The grid was generated using the Receptor Grid Generation tool of Schrödinger, following default settings. The Van der Waals radius scaling used a scaling factor of 1.0 and a partial charge cutoff of 0.25, without applying any per-atom scaling factors or considering aromatic hydrogen and halogen hydrogen bonds. The grid was generated at the site

defined by the SiteMap tool, without incorporating any constraints, rotatable groups, or excluding volumes.

Synthesis of 1-(4-Chloro-3-trifluoromethylphenyl)-3-(4-hydroxyphenyl) urea (DAU)

To a stirred solution of 1-chloro-4-isocyanato-2-(trifluoromethyl) benzene (Sigma-Aldrich) [200 mg, 0.9 mmol] in DCM (4 mL), 4 -aminophenol (Sigma-Aldrich) [100 mg, 0.9 mmol] was added and allowed to stir at room temperature overnight. After completion of the reaction, a white color precipitate was formed. The precipitate was filtered and washed with DCM to remove unreacted starting materials. Then, the precipitate was dried under vacuum to get the product with a yield of 74 %. MS-ESI and HPLC were performed to detect the mass and purity of the product respectively and was found to be high purity with mass matching that of DAU. MS-ESI ($C_{14}H_{10}ClF_3N_2O_2$): calculated for [M+H] +, m/z 331.0383, measured value 331.08.

StkP phosphorylation in *E. coli*

E. coli BL21(DE3) cells transformed with KDC2 plasmid construct were grown as primary culture in Luria Bertani (LB) broth supplemented with 100 µg/mL of ampicillin at 30ºC at 200 rpm for 16 h. For the secondary culture, 1% primary suspension was added to LB broth supplemented with 100 µg/mL of ampicillin. Sorafenib and equivalent amount of DMSO was added into the secondary culture at the specified concentrations and incubated at 37ºC at 200 rpm for 4 h. The bacterial pellet was washed with ice cold PBS and lysed on ice using RIPA buffer supplemented with Protease Inhibitor Cocktail and PhosSTOP for 2-3 h with periodic gentle vortex. The lysates were then sonicated for 2 min time with 2 s on and 3 s off cycles at 30% amplitude, followed by centrifugation at 16,000g for 30 min at 4ºC. The supernatant was collected and stored in 4ºC for further analysis. Total protein of the supernatant was estimated by BCA Protein Assay Kit (Pierce) and 20 µg of the total protein was resolved on a 10% SDS-PAGE. Coomassie stained gel was used as the loading control. Western blotting with anti-Phospho-Threonine monoclonal antibody was performed as described earlier, but with washes using TBS buffer containing 0.5% Tween-20. BL21(DE3) cells transformed with empty pET-22b(+) vector and KDC2 transformed cells induced with 0.5 mM IPTG at OD_{600} of 0.5 for 2 h were used as the negative and positive controls for StkP-KD respectively. Only 2 µg of the clarified lysate was loaded for StkP-KD induced sample to minimize band saturation upon StkP induction.

PrestoBlue cytotoxicity assay

A549 cells were seeded in a 96-well plate (seeding density 10,000 cells/well) and incubated overnight at 37°C and 5% CO_2. The media was removed and 100 µL DMEM supplemented with 10% FBS and 1% penicillin-streptomycin was added. The cells were treated with sorafenib and DMSO at range of concentrations between 2.5-10 µM and incubated for 3 h. 5 µM staurosporine was used as a positive control to induce cell death by apoptosis. After the incubation time, the media was removed, and two PBS washes were given. Fresh 90 µL DMEM complete media was added along with 10 µL of PrestoBlue HS reagent (Invitrogen) and incubated for 10 min. The fluorescence was measured in a Varioskan LUX multimode microplate reader (ThermoFisher) at 560 nm excitation / 590 nm emission. The percentage viability was calculated by normalizing to the untreated control.

Flow cytometry of bacterial viability by Propidium Iodide staining

Propidium Iodide (PI) staining for quantifying dead bacterial cells was performed following the live/dead staining assay protocol with minor modifications. Briefly, ~5×10^7 bacterial cells were harvested post-treatment with either sorafenib or DMSO, washed once with PBS, and resuspended in 500 µL PBS. To this, 1.5 µL of PI dye was added, and the suspension was incubated in the dark for 20 min at room temperature. Following incubation, cells were washed twice with PBS and fixed using 4% paraformaldehyde (PFA, HiMedia) for 10 min at room temperature. Fixed cells were centrifuged and resuspended in PBS for flow cytometric analysis. As a positive control for cell death, bacteria were treated with 70% isopropanol for 20 min at room temperature with shaking at 150 rpm prior to staining. Flow cytometry was performed using a CytoFLEX S Flow Cytometer (Beckman-Coulter), detecting PI-positive cells in the ECD channel (Texas Red).

References cited in this supplementary file:

1. Arang N, Kain HS, Glennon EK, Bello T, Dudgeon DR, Walter ENF, Gujral TS, Kaushansky A. 2017. Identifying host regulators and inhibitors of liver stage malaria infection using kinase activity profiles. Nat Commun 8:1232.
2. Sastry GM, Adzhigirey M, Day T, Annabhimoju R, Sherman W. 2013. Protein and ligand preparation: parameters, protocols, and influence on virtual screening enrichments. J Comput Aided Mol Des 27:221-34.
3. Lu C, Wu C, Ghoreishi D, Chen W, Wang L, Damm W, Ross GA, Dahlgren MK, Russell E, Von Bargen CD, Abel R, Friesner RA, Harder ED. 2021. OPLS4: Improving Force Field Accuracy on Challenging Regimes of Chemical Space. J Chem Theory Comput 17:4291-4300.
4. Johnston RC, Yao K, Kaplan Z, Chelliah M, Leswing K, Seekins S, Watts S, Calkins D, Chief Elk J, Jerome SV, Repasky MP, Shelley JC. 2023. Epik: pK(a) and Protonation State Prediction through Machine Learning. J Chem Theory Comput 19:2380-2388.
5. Halgren TA. 2009. Identifying and characterizing binding sites and assessing druggability. J Chem Inf Model 49:377-89.
